# Six New Methyl Apiofuranosides from the Bark of *Phellodendron chinense* Schneid and Their Inhibitory Effects on Nitric Oxide Production

**DOI:** 10.3390/molecules24101851

**Published:** 2019-05-14

**Authors:** Peng-Fei Wang, Yan-Ping Li, Li-Qin Ding, Shi-Jie Cao, Li-Ning Wang, Feng Qiu

**Affiliations:** 1 School of Chinese Materia Medica, Tianjin University of Traditional Chinese Medicine, Tianjin 300193, China; 13132166710@163.com (P.-F.W.); anney575@163.com (Y.-P.L.); 2 Tianjin State Key Laboratory of Modern Chinese Medicine, Tianjin University of Traditional Chinese Medicine, Tianjin 300193, China; ruby70303@163.com (L.-Q.D.); haojiejie_1988@126.com (S.-J.C.)

**Keywords:** *Phellodendron chinense* Schneid, methyl apiofuranoside, nitric oxide

## Abstract

A chemical investigation on 70% EtOH extract from the bark of *Phellodendron chinense* Schneid (Rutaceae) led to six new methyl apiofuranosides (**1**–**6**), and ten known compounds (**7**–**16**). All these compounds were characterized by the basic analysis of the spectroscopic data including extensive 1D-, 2D-NMR (HSQC, HMBC), and high-resolution mass spectrometry, and the absolute configurations were determined by both empirical approaches and NOESY. Inhibitory effects of compounds **1**–**9** and **11**–**16** on nitric oxide production were investigated in lipopolysaccharide (LPS)-mediated RAW 264.7 cells, as a result, most of these isolates inhibited nitric oxide (NO) release, and among them **9**, **11,** and **12** displayed the strongest inhibition on NO release at the concentration of 12.5 μM.

## 1. Introduction

**Figure 1:**
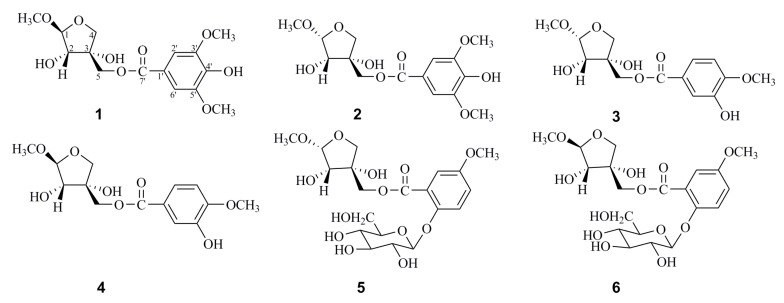
Structures of compounds **1**–**6**.

*Phellodendron chinense* Schneid is a deciduous tree also known as “Huang Bai” and found widely in the east and northeast of Asia [1]. The genus *Phellodendron*, belonging to the family Rutaceae, comprises of approximately 10 species. Among them, the bark of *P. chinense* Schneid, well-known as an oriental folk medicine, has been used for the treatment of meningitis, bacillary dysentery, pneumonia, tuberculosis, and liver cirrhosis for centuries, and is also an important natural source of berberine [2,3]. Previous phytochemical studies on this plant revealed the presence of alkaloids, which mainly belong to isoquinoline alkaloids, berberine-type, and aporphine-type [4]. In addition, some flavonoids, triterpenoids, and coumarins were also reported [5,6]. Pharmacology studies have demonstrated that its crude extracts and active compounds possess wide pharmacological activities, especially hypoglycemic, anti-inflammatory, and antibacterial activities [7,8]. The reports of methyl apiofuranosides are relatively rare [9,10]; during the course of our investigation on the chemical components of *P. chinense* Schneid, six new methyl apiofuranosides (**1**–**6**) (Figure 1) and ten phenolic acids (**7**–**16**) were obtained. In this report, their isolation, structural characterization, and inhibitory effects on nitric oxide production are described.

## 2. Results

**Figure 2:**
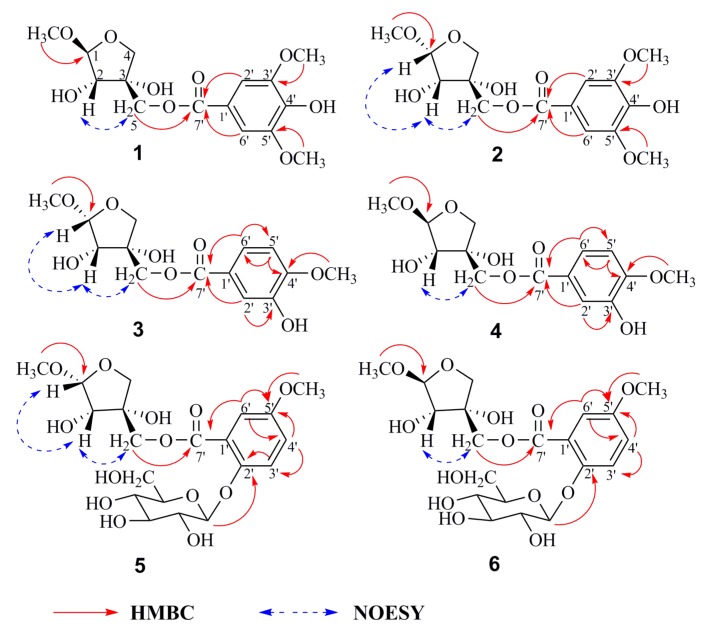
Key HMBC and NOESY correlations of compounds **1**–**6**.

Methyl 5-*O*-(4′-hydroxy-3′,5′-dimethoxy-benzoyl)-β-d-erythro-apiofuranoside (**1**), afforded a molecular formula of C_15_H_20_O_9_ based on the (−)-high-resolution electrospray ionization mass spectra (HRESIMS) ion peak at *m*/*z* 343.1032 [M − H]^−^ (calcd for C_15_H_19_O_9_, 343.1029), indicating six degrees of hydrogen deficiency. The characteristic IR absorptions demonstrated the presence of hydroxy (3412 cm^−1^) and benzene ring (1621, 1112, and 615 cm^−1^) groups. The ^1^H-NMR spectrum of **1** exhibited typical signals for three methoxyl groups (*δ*_H_ 3.89, s, 3H; 3.89, s, 3H; 3.37, s, 3H) and one set of protons of the aromatic system (*δ*_H_ 7.35, 2H, s) (Table 1). In addition, signals in the region of *δ*_H_ 3.50–5.50 mainly came from one sugar unit, characterized by an anomeric proton signal at *δ*_H_ 4.86 (1H, d, *J* = 2.5 Hz). In the ^13^C-NMR spectrum, apart from six characteristic carbon signals for a benzene ring at *δ*_C_ 121.2, 108.5, 149.1, 142.3, 149.1, and 108.5, nine carbon resonances were observed and ascribed to one ester carbonyl at *δ*_C_ 167.9, five oxygen bearing carbons at *δ*_C_ 111.6, 78.9, 79.1, 75.0, and 67.7 and three methoxy carbons at *δ*_C_ 57.0, 57.0, and 56.0 with the aid of an HSQC experiment. These characteristic signals, in combination with the HRESIMS data, implied that **1** is a methyl apiofuranose [11]. According to previous reports, apiose unit with 1-OH and 2-OH in trans configuration presents constant coupling *J*_1,2_ 0–2 Hz, whereas cis configuration is characterized by *J*_1,2_ 3–4 Hz [12,13]. It should be noted that there was no apiose authentic sample at hand, and this branched chain sugar can occur in four isomeric forms. The β-d-apiofuranose moiety was characterized on the basis of the carbon chemical shift and the coupling constant of the anomeric proton, in combination with the NOE correlation between H-2 and H-5 [9,14,15,16]. The methyl apiose unit was therefore identified as a methyl β-d-apiofuranoside based on the anomeric proton singlet at *δ*_H_ 4.86 (1H, d, *J* = 2.5 Hz), the chemical shift of C-1 at 111.6, and on the NOESY correlation between H-2 (*δ*_H_ 3.97, d, *J* = 2.5 Hz) and H-5 (*δ*_H_ 4.32, s, 2H) by empirical approaches reported by Ishii [9]. Furthermore, the observable HMBC correlations (Figure 2) of 3′-OCH_3_/C-3′; 5′-OCH_3_/C-5′; H-2′ and H-6′/C-7′; 1-OCH_3_/C-1 were used to establish 4′-hydroxy-3′,5′-dimethoxy-benzoyl. HMBC correlations from H-5 (*δ*_H_ 4.32) to the ester carbonyl at *δ*_C_ 167.9 (C=O) suggested that the benzoyl group was attached to C-5 of the methyl apiose unit (Figures S1-1–S1-9). Consequently, the structure of **1** was determined to be methyl 5-*O*-(4′-hydroxy-3′,5′-dimethoxy-benzoyl)-β-d-erythro-apiofuranoside, and elucidated as shown in Figure 1.

The same molecular formula, C_15_H_20_O_9_, for **2** (methyl 5-*O*-(4′-hydroxy-3′,5′-dimethoxy-benzoyl)-α-d-erythro-apiofuranoside) was established based on its (−)-HRESIMS ion peak at *m*/*z* 343.1031 (calcd for C_15_H_19_O_9_, 343.1029). Analysis of the NMR spectroscopic data suggests that **2** shares the same methyl apiofuranoside skeleton as **1**, except for two differences in the chemical shift of C-1 and *J*_1,2_. Their respective chemical shift of C-1 exhibited 104.7 in **2** but 111.6 in **1**, furthermore, *J*_1,2_ = 4.6 Hz in **2** and *J*_1,2_ = 2.5 Hz in **1** (Table 1). The methyl apiose unit was therefore identified as a methyl α-d-apiofuranoside based on the anomeric proton singlet at *δ*_H_ 4.90 (1H, d, *J* = 4.6 Hz), the chemical shift of C-1 at *δ*_C_ 104.7, and on the NOESY correlation among H-2 (*δ*_H_ 3.99, d, *J* = 4.6 Hz), H-5 (*δ*_H_ 4.29, m, 2H), and H-1 (*δ*_H_ 4.90, d, *J* = 4.6 Hz) [14]. In comparison of the NOESY spectrum of **2** and **1**, there are correlations between H-2/H-1 and H-5/H-1 in **2**, while H-2/H-5 has the correlation in **1** only (Figures S2-1–S2-9). The structure of compound **2** was proposed as shown.

Methyl 5-*O*-(2′-hydroxyl-5′-methoxyl-benzoyl)-α-d-erythro-apiofuranoside (**3**), afforded a molecular formula of C_14_H_18_O_8_ based on the (−)-HRESIMS ion peak at *m*/*z* 313.0921 [M − H]^−^ (calcd for C_14_H_17_O_8_, 313.0923), corresponding to six degrees of hydrogen deficiency. The characteristic IR absorptions demonstrated the presence of hydroxy (3415 cm^−1^) and benzene ring (1604 and 1458 cm^−1^) groups. The ^1^H-NMR spectrum indicated that compound **3** had two methoxyl groups (*δ*_H_ 3.90, s, 3H and 3.43, s, 3H) and one set of protons of the aromatic system at *δ*_H_ 7.59 (1H, d, *J* = 1.9 Hz, H-2′), 7.57 (1H, dd, *J* = 8.8, 1.9 Hz, H-6′) and 6.84 (1H, d, *J* = 8.8 Hz, H-5′), revealing three aromatic protons coupled in an ABX pattern. Analysis of ^1^H-NMR and ^13^C-NMR spectroscopic data suggests that **3** shares the same methyl apiofuranose-type skeleton as **2** (Figures S3-1–S3-9, Supporting Information). In addition, the observable HMBC correlations (Figure 2) of 3′-OCH_3_/C-3′; H-5′/C-4′ and C-6′; H-6′/C-5′ and C-7′; and H-2′/C-3′ and C-7′ were used to establish the 2′-hydroxyl-5′-methoxyl-benzoyl moiety. The structure of compound **3** was assigned as shown.

The same molecular formula, C_14_H_18_O_8_, for **4** was established based on its (−)-HRESIMS ion peak at *m*/*z* 313.0927 (calcd for C_14_H_17_O_8_, 313.0923), respectively. Analysis of their NMR spectroscopic data suggests that **4** shares the same methyl apiofuranoside skeleton as **3**, except for two differences in the chemical shift of C-1 and *J*_1,2_. Their respective chemical shift of C-1 exhibited 111.6 in **4** and 104.7 in **3**, *J*_1,2_ = 2.6 Hz in **4** and *J*_1,2_ = 4.6 Hz in **3** (Table 1). In addition, NOESY data facilitated the determination of the absolute configuration of methyl 5-*O*-(3′-hydroxyl-4′-methoxyl-benzoyl)-β-d-erythro-apiofuranoside (Figures S4-1–S4-9).

Methyl 5-*O*-(2′-*O*-β-d-glucosyl-5′-methoxyl-benzoyl)-α-d-erythro-apiofuranoside (**5**) afforded a molecular formula of C_20_H_28_O_13_ based on the HRESIMS ion peak at *m*/*z* 521.1513 [M + COOH]^−^ (calcd for C_21_H_29_O_15_, 521.1506), corresponding to seven degrees of hydrogen deficiency. Its ^1^H-NMR data revealed the signals for two methoxyl groups (*δ*_H_ 3.90, s, 3H; 3.43, s, 3H), one set of protons of the aromatic system at *δ*_H_ 7.64 (1H, d, *J* = 2.0 Hz, H-6′), 7.68 (1H, dd, *J* = 8.5, 2.0 Hz, H-4′) and 7.22 (1H, d, *J* = 8.5 Hz, H-3′) revealing three aromatic protons coupled in an ABX pattern, two sugar anomeric proton signals at *δ*_H_ 4.90 (1H, d, *J* = 4.5 Hz) and 5.03 (1H, d, *J* = 7.6 Hz) (Table 2). The ^13^C-NMR spectroscopic data suggests that the presence of an ester carbonyl, a benzene ring, a methyl apioforanose, a glucose and two methoxyl groups in **5**, with the aid of an HSQC experiment. The acid hydrolysis of **5** followed by derivatization with l-cysteine methyl ester liberated d-glucose, which were identified by applying HPLC systems equipped with UV detectors and C_18_ reversed-phase columns [17,18]. In addition, the observable HMBC correlations (Figure 2) of H-1′′/C-2′; H-6′/C-4′, C-5′, C-7′; H-3′/C-2′, C-4′; H-4′/C-3′, C-5′; 5′-OCH_3_/C-5′ and H-5/C-7′ were used to establish 2′-*O*-β-D-glucosyl-5′-methoxyl-benzoyl. Similarly, the configuration of **5** was deduced by the analysis of the NOESY spectrum (Figures S5-1–S5-9).

The HRESIMS of **6** displayed an ion peak at *m*/*z* 521.1516 [M + COOH]^−^ (calcd for C_21_H_29_O_15_, 521.1506), suggesting that this compound shares the same molecular formula (C_20_H_28_O_13_) as that of **5**. The resonances in its NMR data (Figures S4-4–S4-7) demonstrated the planar structure of **4** was similar to that of **5** except for two differences in the chemical shift of C-1 and *J*_1,2_. However, the NOESY correlations among H-2 (*δ*_H_ 3.96, d, *J* = 2.6 Hz) and H-5 (*δ*_H_ 4.33, m, 2H) were indicative of a β-d-erythro-apiofuranoside in **6**, which was opposite that of **5**, and further supported by the ^1^H-NMR and downfield-shifted resonance of H-1 at *δ*_H_ 4.86 (1H, d, *J* = 2.6 Hz) and chemical shift of C-1 exhibited 111.6 in ^13^C-NMR (Figures S6-1–S6-9). The structure of compound **6** (methyl 5-*O*-(2′-*O*-β-d-glucosyl-5′-methoxyl-benzoyl)-β-d-erythro-apiofuranoside) was proposed as shown.

Ten known compounds were identified as *p*-coumaric acid (**7**) [19], trans-ferulic acid (**8**) [20], 3,4-dimethoxycinnamic acid (**9**) [21], methyl-*p*-coumarate (**10**) [22], caffeic acid methyl ester (**11**) [23], ferulic acid methyl ester (**12**) [24], (−)-5-*O*-feruloylquinic acid methyl ester (**13**) [25], methyl 4-hydroxybenzoate (**14**) [26], ethyl 3,4-dihydroxybenzoate (**15**) [27], and 4-hydroxy-3,5-dimethoxy benzoic acid methyl ester (**16**) [28] based on their obtained spectroscopic data.

**Figure 3:**
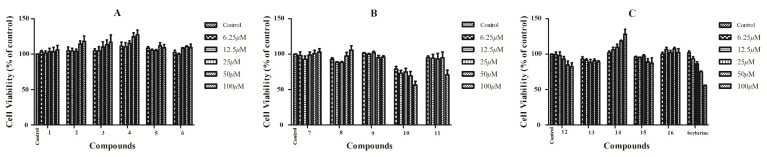
Effects of compounds 1–6 (**A**), compounds 7–11 (**B**), compounds 12–16 and Berberine (**C**) on RAW 246.7 cell viability (6.25–100 μM) compared to the control group (without compound group). Data are represented as mean ± SD of three independent experiments.

**Figure 4:**
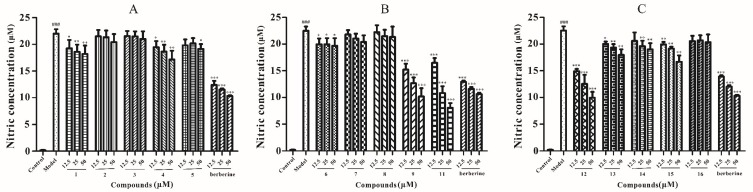
Nitric oxide (NO) inhibitory activity of compounds 1–5 (**A**), compounds 6–9, 11 (**B**) and compounds 12–16 (**C**) on RAW 264.7 cell (12.5 μM, 25 μM, 50 μM). ^###^ *p* < 0.001, compared to the control group (without lipopolysaccharide (LPS)-treated group); * *p* < 0.05, ** *p* < 0.01, and *** *p* < 0.001 compared to the model group (with LPS-treated group). Data are represented as mean ± SD of three independent experiments.

Nitric oxide (NO) is a diatomic free radical that is extremely short lived in biological systems. It is generated from l-arginine by nitric oxide synthase (NOS) and plays an important role in the regulation of physiological responses. The generation of NO is closely associated with inflammation, tumors, and immunoregulation [29,30,31]. In this study, all compounds isolated from the bark of *P. chinense* Schneid were examined for their inhibitory effects on NO production induced by lipopolysaccharide (LPS) in RAW 264.7 cells. In order to exclude the inhibition of NO production caused by cell cytotoxicity, cell viability was evaluated by the MTT method [32]. RAW 264.7 cell was treated with various concentrations of isolates for 24 h and the cell viability was tested by MTT assay as described in Section 2. As shown in Figure 3, the results revealed that no obvious cytotoxicity (over 85% cell survival) for most of compounds at the concentrations range of 6.25–100 μM was observed except for compound **10**. Thus, the NO levels were detected in the RAW 264.7 cells after treated with 12.5 μM, 25 μM, and 50 μM of the tested compounds in subsequent experiments. Most of the isolated compounds inhibited NO release, as shown in Figure 4, and among them methyl apiofuranosides **1, 4,** and **6** exhibited the inhibition on NO release at the concentration of 12.5 μM, while **2**, **3,** and **5** did not affect NO production. Meanwhile, compounds **9**, **11,** and **12** displayed the strongest inhibition on NO release, compared with the positive control berberine [33]. Together, three new methyl apiofuranosides including **1**, **4**, and **6** need to be further investigated on the pharmacological properties of NO inhibition. 

## 3. Materials and Methods

### 3.1. General Experimental Procedure

Optical rotations were measured with a AUTOPOL Ⅳ polarimeter (Rudolph, Hackettstown, NJ, USA). The UV spectra were determined by a UV-2450 visible spectrophotometer (Shimadzu, Tokyo, Japan). Infrared spectra were collected using KBr disks on a Tensor 27 infrared spectrometer (Bruker Beijing Scientific Tech Co., Beijing, China). NMR spectra were recorded on a Bruker ARX-600 spectrometer (600 MHz for ^1^H and 150 MHz for ^13^C, Bruker Beijing Scientific Tech Co., Beijing, China) in CD_3_OD with tetramethylsilane as an internal standard. Chemical shifts were expressed in *δ* (ppm), and coupling constants (*J*) were reported in Hz. High-resolution electrospray ionization mass spectra (HRESIMS) were acquired on a Waters Xevo G2-S UPLC-Q/TOF mass spectrometer (Water, Milford, MA, USA). Preparative HPLC was performed with an ODS column (C-18, 250 × 20 mm, Inertsil Pak, Tokyo, Japan) in a Waters 600 liquid chromatograph apparatus equipped with a Waters 490 UV detector (Water, Milford, MA, USA). Methanol was HPLC grade. Sigel 60 (Qingdao Haiyang Chemical Co., Ltd., Qingdao, China), Sephadex LH-20 (Advanced Technology Industrial Co., Ltd., Hongkong, China), and ODS (40–75 µm, FujiSilysia Chemical Ltd., Kyoto, Japan) were used as column chromatography stationary phases. TLC was carried out on a silica gel 60 plate 20 × 20 cm (Merck, Berlin, Germany). RP-HPLC was performed on an Agilent 1260 Series instrument with an RP-C_18_ column (20 × 200 mm i.d., Shim-pack, Shimadzu, Tokyo, Japan).

### 3.2. Plant Material

The dried bark of *P. chinense* Schneid was collected in Anguo, Hebei Province, People’s Republic of China. A voucher specimen (AP-2014-62) was identified by Professor Li-Juan Zhang and deposited at the School of Chinese Materia Medica, Tianjin University of Traditional Chinese Medicine.

### 3.3. Extraction and Isolation

The plant material (9.0 kg) was cut into small pieces and heated at reflux with 70% aqueous EtOH (3 × 90 L). The resulting EtOH extract was concentrated in vacuo at 40 °C, suspended in H_2_O (5 L). A total of 4% dilute sulfuric acid adjusted the pH to 4–5, followed by enrichment of alkaloids by 732 cation-exchange resins to obtain alkaloid fraction (20 g) and non-alkaloid fraction (435 g). The non-alkaloids were detected by TLC, and the Dragendorff’s reagent reaction was negative. The non-alkaloid fraction (435 g) was first fractionated by macroporous adsorptive resin D101, eluted with a step gradient system of EtOH/H_2_O (*v*/*v*, 0–100%), to afford six subfractions (N1–N6). The N5 (32 g) was subjected to silica gel column chromatography (10 cm × 120 cm) using a gradient mixture of CH_2_Cl_2_-MeOH (100:0, 100:1, 70:1, 50:1, 30:1, 10:1, 5:1, 1:1, 0:100) as eluent to give eight fractions (N51–N58). N51 was eluted with CH_2_Cl_2_-MeOH (1:1) on Sephadex LH-20 and then further purified by ODS column chromatography eluted with MeOH/H_2_O (20:80, 50:50, 80:20) and by repeated RP-18 HPLC preparation to give **1** (11.0 mg), **2** (22.0 mg), **3** (10.6 mg), **4** (8.6 mg), **5** (9.6 mg), and **6** (10.4 mg).

*Methyl 5-O-(4′-hydroxy-3′,5′-dimethoxy-benzoyl)-β-d-erythro-apiofuranoside* (**1**): colorless or white acicular crystal; ${\left[\alpha \right]}_{D}^{25}$−24° (*c* 0.1, MeOH); UV (MeOH) *λ*_max_ (log *ε*) 216 (4.3), 279 (4.0) nm; IR (KBr) *ν*_max_ 3412, 2968, 1621, 1112, 1054, 1024, 615 cm^−1^; ^1^H and ^13^C-NMR data (Table 1); HRESIMS *m*/*z* 343.1032 [M − H]^−^ (calcd for C_15_H_19_O_9_, 343.1029).

*Methyl 5-O-(4′-hydroxy-3′,5′-dimethoxy-benzoyl)-α-d-erythro-apiofuranoside* (**2**): colorless oil; ${\left[\alpha \right]}_{D}^{25}$+50° (*c* 0.1, MeOH); UV (MeOH) *λ*_max_ (log *ε*) 217 (4.3), 279 (4.0) nm; IR (KBr) *ν*_max_ 3416, 2965, 1619, 1463, 1363, 1222, 1045, 778, 612 cm^−1^; ^1^H and ^13^C-NMR data (Table 1); HRESIMS *m*/*z* 343.1031 [M − H]^−^ (calcd for C_15_H_19_O_9_, 343.1029).

*Methyl 5-O-(2′-hydroxyl-5′-methoxyl-benzoyl)-α-d-erythro-apiofuranoside* (**3**): colorless oil; ${\left[\alpha \right]}_{D}^{25}$+56° (*c* 0.1, MeOH); UV (MeOH) *λ*_max_ (log *ε*) 204 (4.0), 264 (3.8) nm; IR (KBr) *ν*_max_ 3415, 2941, 1707, 1604, 1275, 1059, 768 cm^−1^; ^1^H and ^13^C-NMR data (Table 1); HRESIMS *m*/*z* 313.0921 [M − H]^−^ (calcd for C_14_H_17_O_8_, 313.0923).

*Methyl 5-O-(3′-hydroxyl-4′-methoxyl-benzoyl)-β-d-erythro-apiofuranoside* (**4**): colorless oil; ${\left[\alpha \right]}_{D}^{25}$−6° (*c* 0.1, MeOH); UV (MeOH) *λ*_max_ (log *ε*) 204 (4.0), 264 (3.8) nm; IR (KBr) *ν*_max_ 3414, 2938, 2351, 1717, 1522, 1467, 1324, 1048, 616 cm^−1^; ^1^H and ^13^C-NMR data (Table 1); HRESIMS *m*/*z* 313.0927 [M − H]^−^ (calcd for C_14_H_17_O_8_, 313.0923).

*Methyl 5-O-(2′-O-β-d-glucosyl-5′-methoxyl-benzoyl)-*α*-d-erythro-apiofuranoside* (**5**): colorless oil; ${\left[\alpha \right]}_{D}^{25}$+14° (*c* 0.1, MeOH); UV (MeOH) *λ*_max_ (log *ε*) 204 (4.2), 257 (3.8) nm; IR (KBr) *ν*_max_ 2947, 2351, 1518, 1463, 1375, 1321, 1025, 668 cm^−1^; ^1^H and ^13^C-NMR data (Table 2); HRESIMS *m*/*z* 521.1513 [M + COOH]^−^ (calcd for C_21_H_29_O_15_, 521.1506).

*Methyl 5-O-(2′-O-β-d-glucosyl-5′-methoxyl-benzoyl)-β-d-erythro-apiofuranoside* (**6**): colorless oil; ${\left[\alpha \right]}_{D}^{25}$−24° (*c* 0.1, MeOH); UV (MeOH) *λ*_max_ (log *ε*) 204 (4.2), 257 (3.8) nm; IR (KBr) *ν*_max_ 2962, 2351, 1701, 1522, 1467, 1048, 669 cm^−1^; ^1^H and ^13^C-NMR data (Table 2); HRESIMS *m*/*z* 521.1516 [M + COOH]^−^ (calcd for C_21_H_29_O_15_, 521.1506).

### 3.4. Sugar Identification

Compounds **5** and **6** (1.0 mg each) were each hydrolyzed with 2.0 M HCl (4.0 mL), heated for 1.0 h at 85 °C and extracted with ethyl acetate, and the reaction mixture was concentrated [17]. The residue was dissolved in pyridine (1 mL) and reacted with l-cysteine methyl ester (4.0 mg) for 1.0 h at 60 °C, and then *ο*-tolyl isothiocyanate (10 µL) was added to the mixture and stirred at 60 °C for another 1.0 h. The derivatives were analyzed by HPLC and detected at 250nm [18]. Mobile phase: MeCN-H_2_O (25:75), flow rate: 1.0 mL/min, t_R_ = 15.54 min (l-glucose), t_R_ = 16.98 min (d-glucose), and the migration time of the derivatives of **5** and **6** was also 16.98 min, which revealed the sugar moiety in compounds **5** and **6** was d-glucose. The d-glucose and l-glucose as reference samples were also derivatized and analyzed applying the same procedures mentioned above.

### 3.5. Cell Culture

The murine RAW 264.7 macrophage cells were obtained from Cell Bank of the Shanghai Institute of Cell Biology and Biochemistry, Chinese Academy of Sciences (Shanghai, China). The cells were cultured in Dulbecco’s modified Eagle’s medium containing 10% fetal bovine serum and penicillin-streptomycin (100 U/mL) at 37 °C with 5% CO_2_.

### 3.6. Cell Viability Assay

An MTT assay was used to evaluate RAW 264.7 cell viability as previously described [34]. The mitochondrial-dependent reduction of 3-(4,5-dimethylthizaol-2yl)-2,5-diphenyl tetrazolium bromide (MTT) to formazan was used to measure cell respiration as an indicator of cell viability [35]. Briefly, after 24 h incubation with or without compounds (6.25–100 μM), an MTT solution (final concentration 5 mg/mL) was added and the cells were incubated for another 2.5 h at 37 °C. After removing the supernatant, 150 μL of DMSO was added to the cells to dissolve the formazan. The absorbance of each group was measured by using a microplate reader at a wavelength of 490 nm. Results are expressed as a percentage of viable cells when compared with the control group. The viability of RAW 264.7 cells of the control group (with 0.1%DMSO only) is defined as 100%.

### 3.7. Measurement of NO Release

The accumulated nitrite in the supernatant was evaluated using the Griess reagent [36,37] (Beyotime, Nanjing, China). RAW 264.7 macrophage cells were seeded in 96-well culture plates (5 × 10^4^ cells/well) for 24 h. Then, various concentrations of test compounds were added, after 2.5 h incubation, with the presence of 1 μg/mL LPS. 0.1% DMSO and 1 μg/mL LPS without test compounds were added as model group, only with the presence of 0.1% DMSO and equivalent DMEM as the control group. Twenty hours later, culture supernatant and Griess reagent were mixed and then incubated for 10 min. The optical density of the mixture was read at 540 nm using an automated microplate reader. The NO inhibitory rate was measured in relation to the model group (cells were treated with LPS only). Berberine (purity ≥98%, Yuanye, Shanghai, China) served as the positive control.

## Figures and Tables

**Table 1:** ^1^H-NMR (600 MHz) and ^13^C-NMR (150 MHz) spectroscopic data for compounds **1**–**4**.

	1 ^a^		2 ^a^		3 ^a^		4 ^a^	
Position	*δ*_C_	*δ*_H_ (*J* in Hz)	*δ*_C_	*δ*_H_ (*J* in Hz)	*δ*_C_	*δ*_H_ (*J* in Hz)	*δ*_C_	*δ*_H_ (*J* in Hz)
Api-1	111.6	4.86 (1H, d, *J* = 2.5 Hz)	104.7	4.90 (1H, d, *J* = 4.6 Hz)	104.7	4.90 (1H, d, *J* = 4.6 Hz)	111.6	4.86 (1H, d, *J* = 2.6 Hz)
2	78.9	3.97 (1H, d, *J* = 2.5 Hz)	74.5	3.99 (1H, d, *J* = 4.6 Hz)	74.4	3.99 (1H, d, *J* = 4.6 Hz)	78.8	3.96 (1H, d, *J* = 2.6 Hz)
3	79.1		76.6		76.7		79.1	
4	75.0	4.04 (1H, d, *J* = 9.8 Hz)	75.4	4.07 (1H, d, *J* = 9.9 Hz)	75.4	4.07 (1H, d, *J* = 9.8 Hz)	75.0	4.03 (1H, d, *J* = 9.8 Hz)
		3.89 (1H, d, *J* = 9.8 Hz)		3.91 (1H, d, *J* = 9.9 Hz)		3.91 (1H, d, *J* = 9.8 Hz)		3.89 (1H, d, *J* = 9.8 Hz)
5	67.7	4.32 (2H, m)	68.7	4.29 (2H, m)	68.4	4.28 (2H, m)	67.5	4.31 (2H, m)
1′	121.2		121.2		125.4		125.4	
2′	108.5	7.35 (1H, s)	108.5	7.35 (1H, s)	116.1	7.59 (1H, d, *J* = 1.9 Hz)	116.1	7.59 (1H, d, *J* = 1.9 Hz)
3′	149.1		149.1		148.9		148.9	
4′	142.3		142.3		153.2		153.2	
5′	149.1		149.1		113.9	6.84 (1H, d, *J* = 8.8 Hz)	113.8	6.85 (1H, d, *J* = 8.0 Hz)
6′	108.5	7.35 (1H, s)	108.5	7.35 (1H, s)	122.4	7.57 (1H, dd, *J* = 8.8, 1.9 Hz)	122.4	7.58 (1H, dd, *J* = 8.0, 1.9 Hz)
7′	167.9		167.9		168.0		168.0	
3′-OCH_3_	57.0	3.89 (3H, s)	57.0	3.88 (3H, s)				
4′-OCH_3_					56.6	3.90 (3H, s)	56.6	3.90 (3H, s)
5′-OCH_3_	57.0	3.89 (3H, s)	57.0	3.88 (3H, s)				
1-OCH_3_	56.0	3.37 (3H, s)	56.0	3.43 (3H, s)	55.7	3.43 (3H, s)	55.7	3.38 (3H, s)

^a^ Data were recorded in CD_3_OD.

**Table 2:** ^1^H-NMR (600 MHz) and ^13^C-NMR (150 MHz) spectroscopic data for compounds **5**–**6**.

	5 ^a^		6 ^a^	
Position	*δ*_C_	*δ*_H_ (*J* in Hz)	*δ*_C_	*δ*_H_ (*J* in Hz)
Api-1	104.7	4.90 (1H, d, *J* = 4.5 Hz)	111.6	4.86 (1H, d, *J* = 2.6 Hz)
2	74.5	4.00 (1H, d, *J* = 4.5 Hz)	78.8	3.96 (1H, d, *J* = 2.6 Hz)
3	76.6		79.1	
4	75.4	4.07 (1H, d, *J* = 10.0 Hz)	75.0	4.03 (1H, d, *J* = 9.8 Hz)
		3.92 (1H, d, *J* = 10.0 Hz)		3.90 (1H, d, *J* = 9.8 Hz)
5	68.7	4.31 (2H, m)	67.8	4.33 (2H, m)
1′	125.3		125.3	
2′	152.5		152.5	
3′	116.6	7.22 (1H, d, *J* = 8.5 Hz)	116.6	7.22 (1H, d, *J* = 8.5 Hz)
4′	124.9	7.68 (1H, dd, *J* = 8.5, 2.0 Hz)	124.9	7.67 (1H, dd, *J* = 8.5, 2.0 Hz)
5′	150.6		150.6	
6′	114.5	7.64 (1H, d, *J* = 2.0 Hz)	114.5	7.64 (1H, d, *J* = 2.0 Hz)
7′	167.6		167.5	
5′-OCH_3_	56.9	3.90 (3H, s)	56.9	3.91 (3H, s)
1-OCH_3_	55.7	3.43 (3H, s)	56.0	3.38 (3H, s)
Glc-1′′	102.1	5.03 (1H, d, *J* = 7.6 Hz)	102.1	5.03 (1H, d, *J* = 7.6 Hz)
2′′	74.9	3.53 (1H, m)	75.0	3.53 (1H, m)
3′′	78.6	3.47 (1H, m)	78.5	3.47 (1H, m)
4′′	71.4	3.40 (1H, m)	71.4	3.42 (1H, m)
5′′	78.0	3.49 (1H, m)	78.0	3.49 (1H, m)
6′′	62.6	3.70 (1H, dd, *J* = 12.0, 5.6 Hz)	62.6	3.69 (1H, dd, *J* = 12.0, 5.6 Hz)
		3.87 (1H, m)		3.88 (1H, m)

^a^ Data were recorded in CD_3_OD.

## Supplementary Materials

Supplementary Materials are available online.

## Abbreviations

**DMEM**	dulbecco’s modified eagle medium
**DMSO**	Dimethyl Sulfoxide
**HMBC**	Heteronuclear Multiple Bonding Correlation
**HSQC**	Heteronuclear Single Quantum Correlation
**MTT**	3-(4,5-dimethyl-2-thiazolyl)-2,5-diphenyl-2-H-tetrazolium bromide
**NOE**	Nuclear Overhauser Enhancement
**NOESY**	Nuclear Overhauser Enhancement Spectroscopy
**RAW**	leukemia cellsin mouse macrophage
