# Supplementary material for: Six New Methyl Apiofuranosides from the Bark of Phellodendron chinense Schneid and Their Inhibitory Effects on Nitric Oxide Production
Source: Molecules. 2019 May 14;24(10):1851. doi: 10.3390/molecules24101851 (PMC6572284; doi:10.3390/molecules24101851)
Supplement: Supplementary file 1 [file molecules-24-01851-s001.pdf]

Supplementary Materials

Six New Methyl Apiofuranosides from the Bark of Phellodendron chinense Schneid and Their Inhibitory Effects on Nitric Oxide Production

Peng-Fei Wang [1,2†], Yan-Ping Li [1,2†], Li-Qin Ding [2], Shi-Jie Cao [2], Li-Ning Wang [1 *], Feng Qiu [1,2, *]

[1] School of Chinese Materia Medica, Tianjin University of Traditional Chinese Medicine, Tianjin 300193, China; 13132166710@163.com (P.-F.W.); anney575@163.com (Y.-P.L.);

[2] Tianjin State Key Laboratory of Modern Chinese Medicine, Tianjin University of Traditional Chinese Medicine, Tianjin 300193, China; ruby70303@163.com (L.-Q.D.); haojiejie_1988@126.com (S.-J.C.);

* Correspondence: fengqiu20070118@163.com (F.Q.); lining.wang@tjutcm.edu.cn (L.-N.W.); Tel.: +86-22-5959-6223 (F.Q.); +86-22-59596238 (L.-N.W.)

† These authors contributed equally to this work.

Figure of Contents

Figure S1-1 UV spectrum of compound **1** in CH_3OH …………………………...S5

Figure S1-2 IR spectrum of compound **1** (KBr disc) ……………………………S5

Figure S1-3 HRESIMS of compound **1** …………………………………………S5

Figure S1-4 ^{1}H NMR (600 MHz, CD_3OD) spectrum of compound **1** S6

Figure S1-5 ^{13}C NMR (150 MHz, CD_3OD) spectrum of compound **1** ………....S6

Figure S1-6 HSQC (CD_3OD) spectrum of compound **1** ………………………...S7

Figure S1-7 HMBC (CD_3OD) spectrum of compound **1** ………………………..S7

Figure S1-8 NOESY (CD_3OD) spectrum of compound **1** …………………........S8

Figure S1-9 Key NOESY (CD_3OD) spectrum of compound **1** ……….…..……...S8

Figure S2-1 UV spectrum of compound **2** in CH_3OH ……………….…………S9

Figure S2-2 IR spectrum of compound **2** (KBr disc) …… ……………………….S9

Figure S2-3 HRESIMS of compound **2** ………………… …………………….S9

Figure S2-4 ^{1}H NMR (600 MHz, CD_3OD) spectrum of compound **2** …………...S10

Figure S2-5 ^{13}C NMR (150 MHz, CD_3OD) spectrum of compound **2** …………..S10

Figure S2-6 HSQC (CD_3OD) spectrum of compound **2** …………………………S11

Figure S2-7 HMBC (CD_3OD) spectrum of compound **2** ………………………...S11

Figure S2-8 NOESY (CD_3OD) spectrum of compound **2** ………………………..S12

Figure S2-9 Key NOESY (CD_3OD) spectrum of compound **2** …………………...S12

Figure S3-1 UV spectrum of compound **3** in CH_3OH ……………………………S13

Figure S3-2 IR spectrum of compound **3** (KBr disc) …………………………….S13

Figure S3-3 HRESIMS of compound **3** ………………………………………….S13

Figure S3-4 ^{1}H NMR (600 MHz, CD_3OD) spectrum of compound **3** …………...S14

Figure S3-5 ^{13}C NMR (150 MHz, CD_3OD) spectrum of compound **3** …………..S14

Figure S3-6 HSQC (CD_3OD) spectrum of compound **3** …………………………S15

Figure S3-7 HMBC (CD_3OD) spectrum of compound **3** ………………………...S15

Figure S3-8 NOESY (CD_3OD) spectrum of compound **3** ………………………..S16
Figure S3-9 Key NOESY (CD_3OD) spectrum of compound **3** …………………...S16

Figure S4-1 UV spectrum of compound **4** in CH_3OH ……………………………S17
Figure S4-2 IR spectrum of compound **4** (KBr disc) …………………………….S17
Figure S4-3 HRESIMS of compound **4** ………………………………………….S17
Figure S4-4 ^{1}H NMR (600 MHz, CD_3OD) spectrum of compound **4** …………...S18
Figure S4-5 ^{13}C NMR (150 MHz, CD_3OD) spectrum of compound **4** …………..S18
Figure S4-6 HSQC (CD_3OD) spectrum of compound **4** …………………………S19
Figure S4-7 HMBC (CD_3OD) spectrum of compound **4** ………………………...S19
Figure S4-8 NOESY (CD_3OD) spectrum of compound **4** ………………………..S20
Figure S4-9 Key NOESY (CD_3OD) spectrum of compound **4** …………………...S20

Figure S5-1 UV spectrum of compound **5** in CH_3OH ……………………………S21
Figure S5-2 IR spectrum of compound **5** (KBr disc) …………………………….S21
Figure S5-3 HRESIMS of compound **5** ………………………………………….S21
Figure S5-4 ^{1}H NMR (600 MHz, CD_3OD) spectrum of compound **5** ……….......S22
Figure S5-5 ^{13}C NMR (150 MHz, CD_3OD) spectrum of compound **5** …………..S22
Figure S5-6 HSQC (CD_3OD) spectrum of compound **5** …………………………S23
Figure S5-7 HMBC (CD_3OD) spectrum of compound **5** ………………………...S23
Figure S5-8 NOESY (CD_3OD) spectrum of compound **5** ………………………..S24
Figure S5-9 Key NOESY (CD_3OD) spectrum of compound **5** …………………...S24

Figure S6-1 UV spectrum of compound **6** in CH_3OH ……………………………S25
Figure S6-2 IR spectrum of compound **6** (KBr disc) …………………………….S25
Figure S6-3 HRESIMS of compound **6** ………………………………………….S25
Figure S6-4 ^{1}H NMR (600 MHz, CD_3OD) spectrum of compound **6** …………...S26
Figure S6-5 ^{13}C NMR (150 MHz, CD_3OD) spectrum of compound **6** …………..S26
Figure S6-6 HSQC (CD_3OD) spectrum of compound **6** …………………………S27

Figure S6-7 HMBC (CD_3OD) spectrum of compound **6** …………………………...S27

Figure S6-8 NOESY (CD_3OD) spectrum of compound **6** ………………………..S28

Figure S6-9 Key NOESY (CD_3OD) spectrum of compound **6** ……....…………...S28

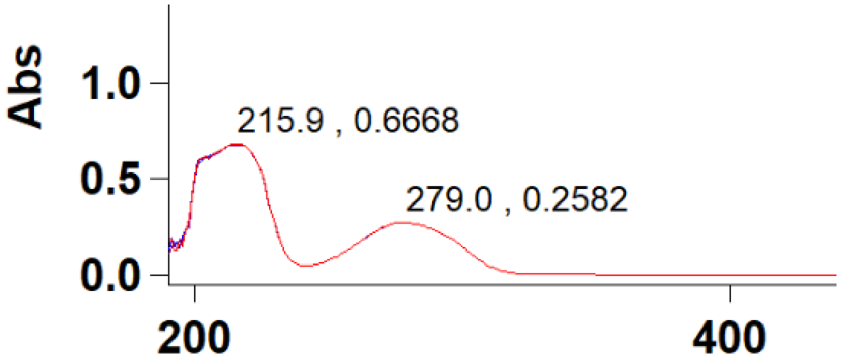

Figure S1-1 UV spectrum of compound **1** in CH_3OH

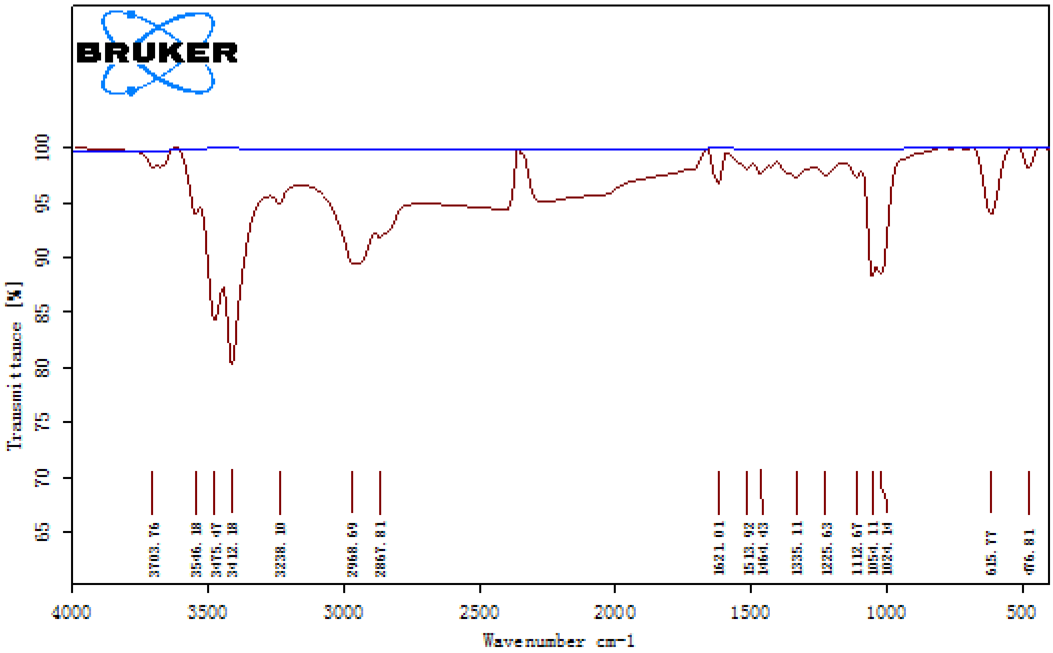

Figure S1-2 IR spectrum of compound **1** (KBr disc)

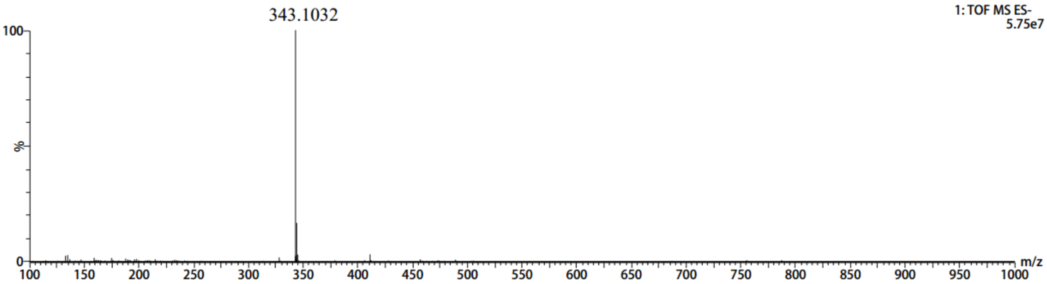

Elemental Composition Calculator

**Target m/z:**	343.1032	**Result type:**	Negative ions	**Species:**	$[M-H]^-$
**Elements:**		C(0-20),H(0-120),O(0-10)			

Ion Formula	Calculated m/z	PPM Error
$C_{15}H_{19}O_{9}$	343.1029	0.87

Figure S1-3 HRESIMS of compound **1**

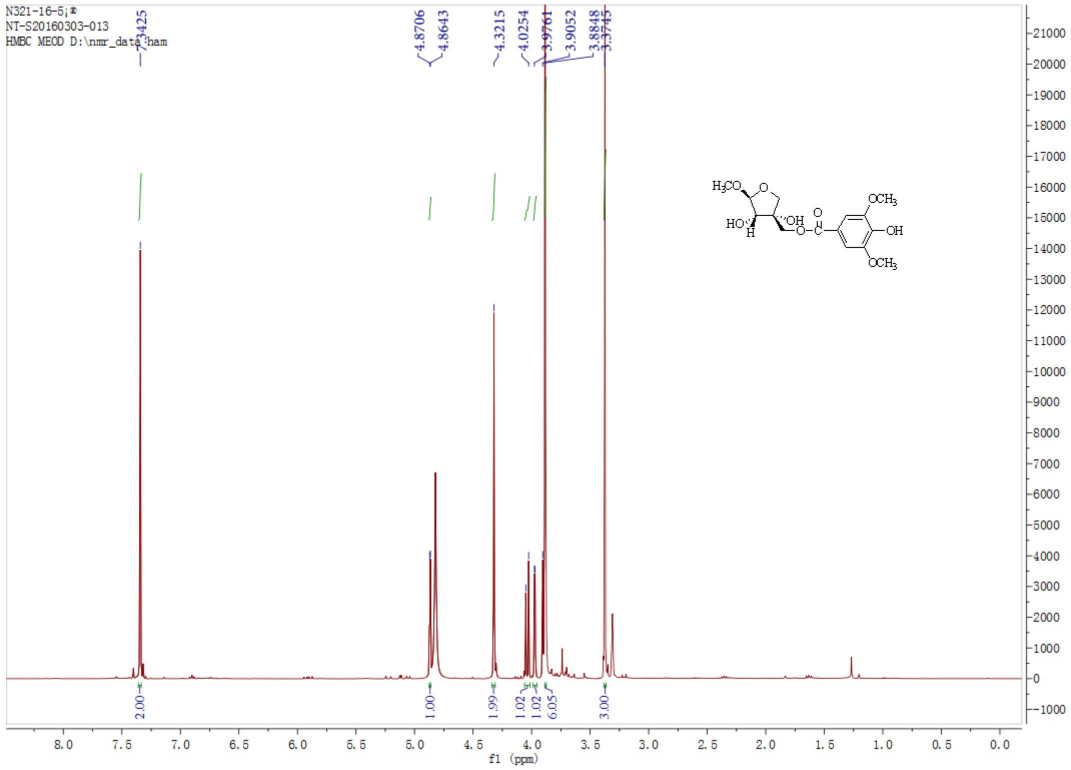

Figure S1-4 ^{1}H NMR (600 MHz, CD_3OD) spectrum of compound **1**

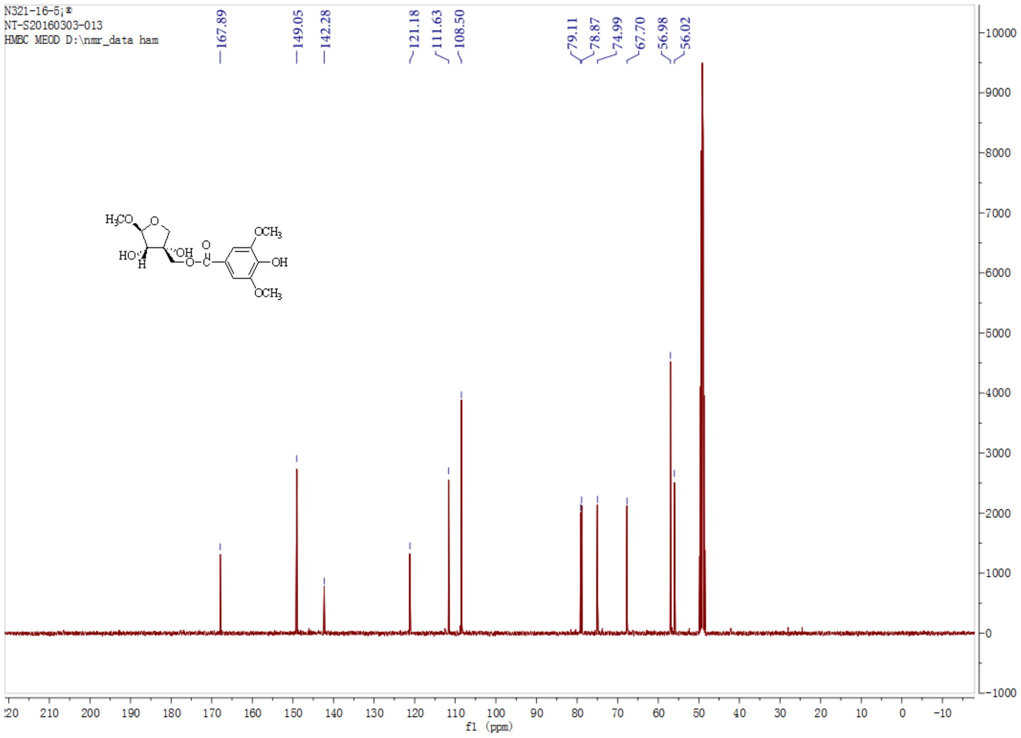

Figure S1-5 ^{13}C NMR (150 MHz, CD_3OD) spectrum of compound **1**

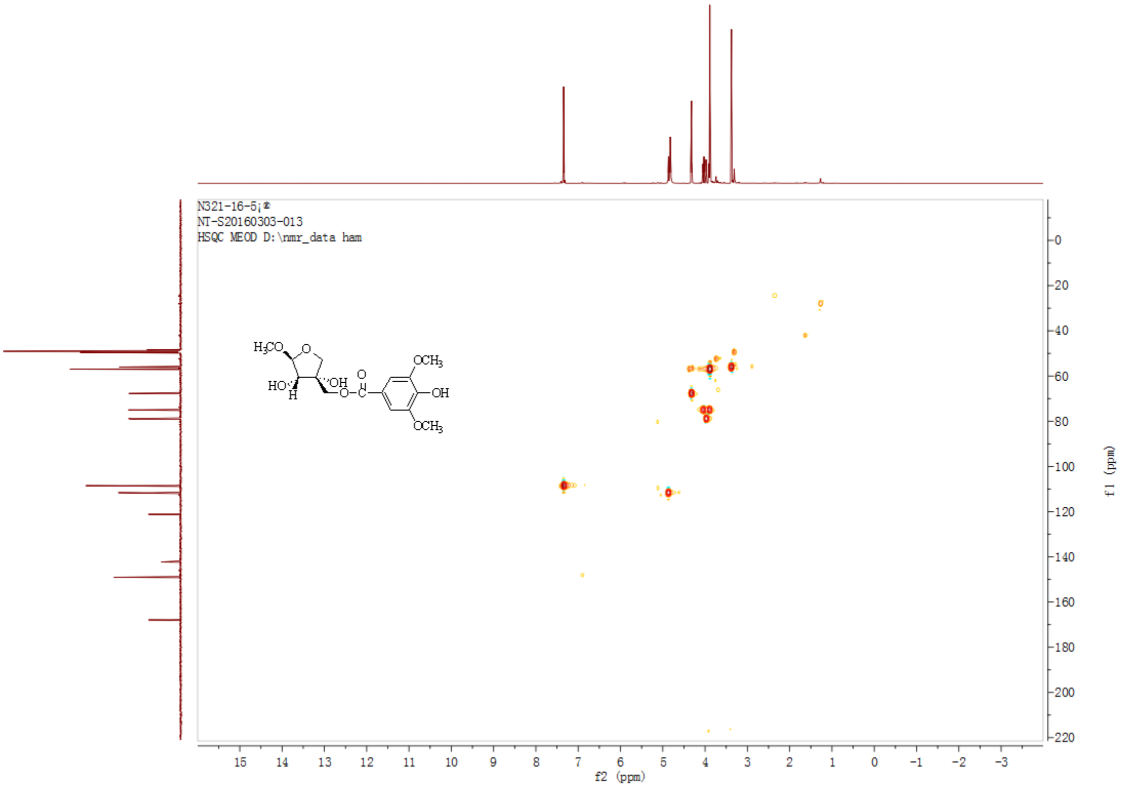

Figure S1-6 HSQC (CD_3OD) spectrum of compound **1**

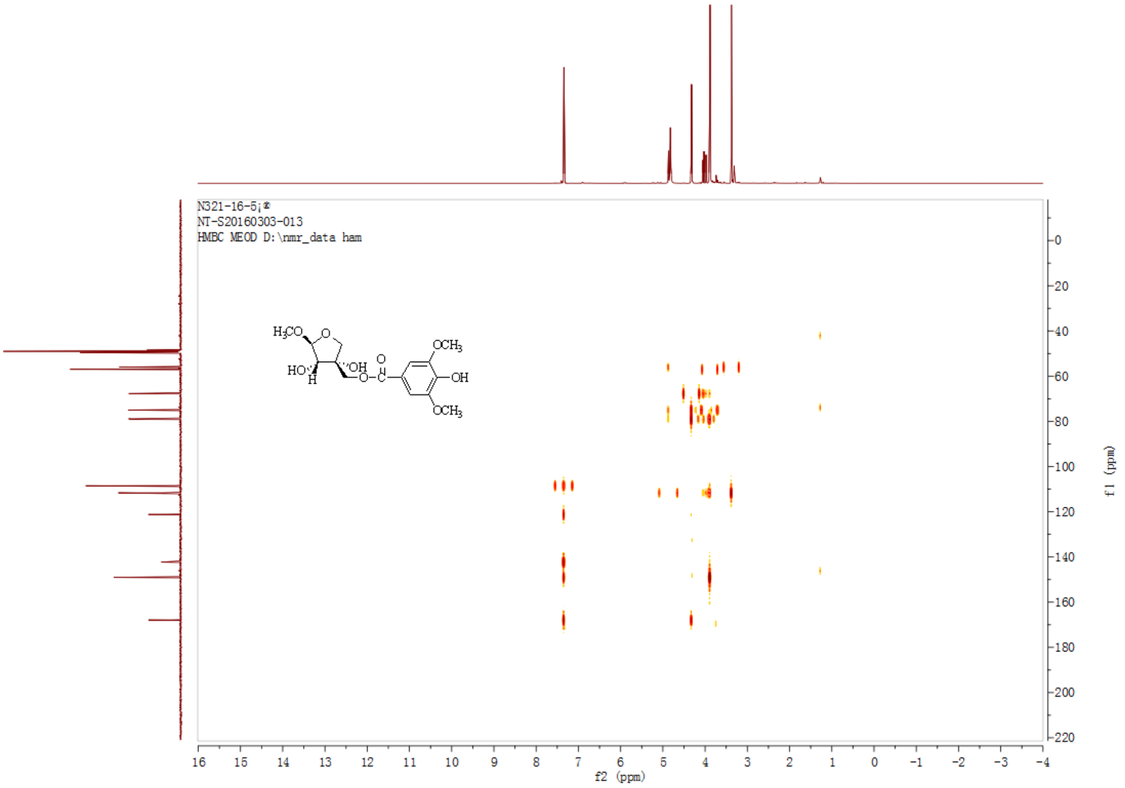

Figure S1-7 HMBC (CD_3OD) spectrum of compound **1**

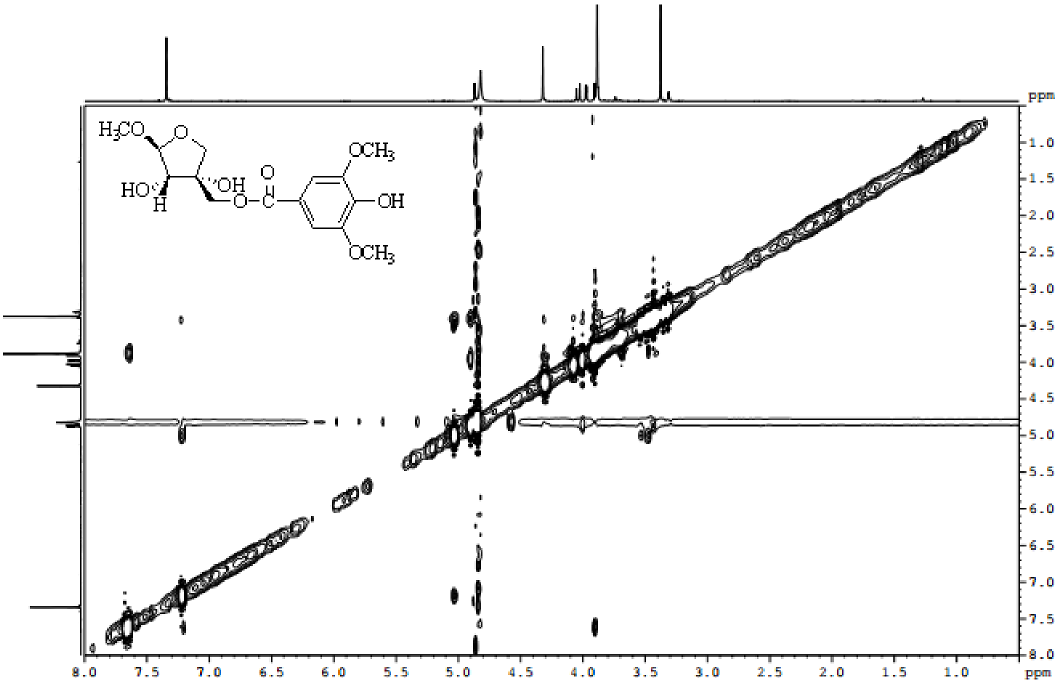

Figure S1-8 NOESY (CD_3OD) spectrum of compound **1**

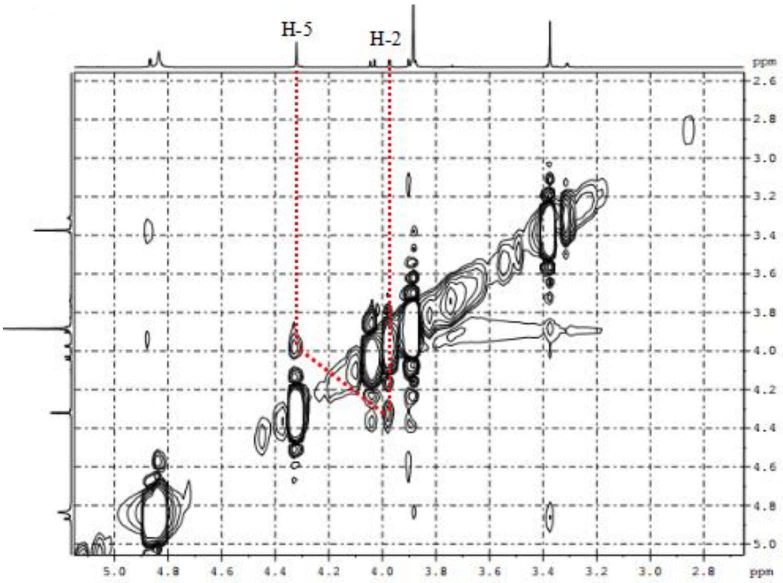

Figure S1-9 Key NOESY (CD_3OD) spectrum of compound **1**

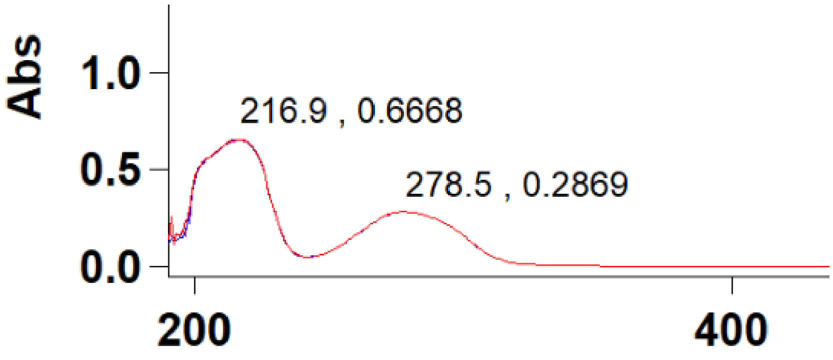

Figure S2-1 UV spectrum of compound **2** in CH_3OH

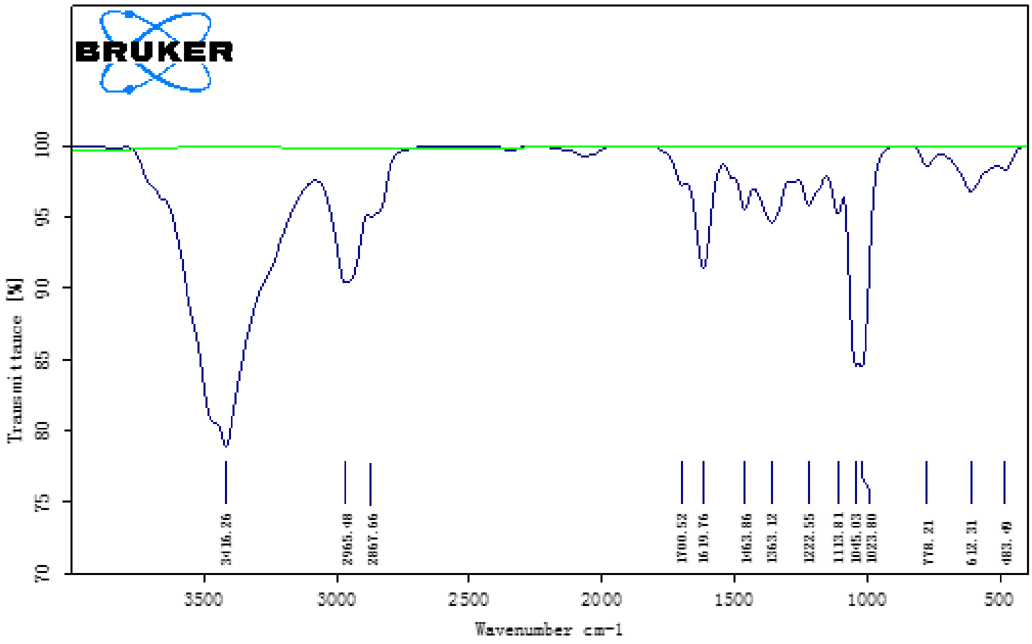

Figure S2-2 IR spectrum of compound **2** (KBr disc)

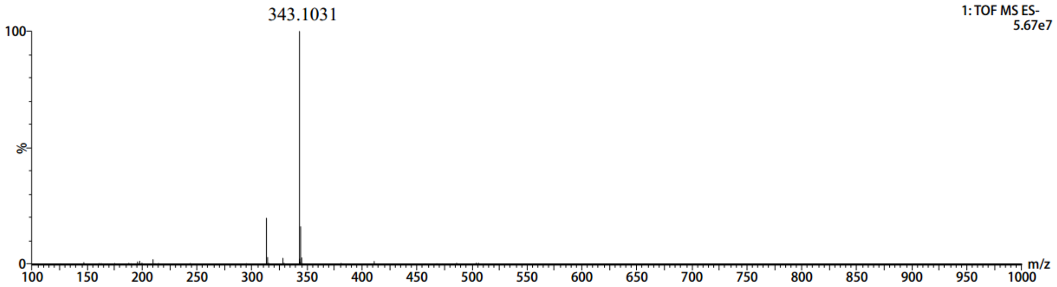

Ele

mental Composition Calculator

**Target m/z:**	343.1031	**Result type:**	Negative ions	**Species:**	$[M-H]^-$
**Elements:**		C(0-20),H(0-120),O(0-10)			

Ion Formula	Calculated m/z	PPM Error
$C_{15}H_{19}O_9$	343.1029	0.58

Figure S2-3 HRESIMS of compound **2**

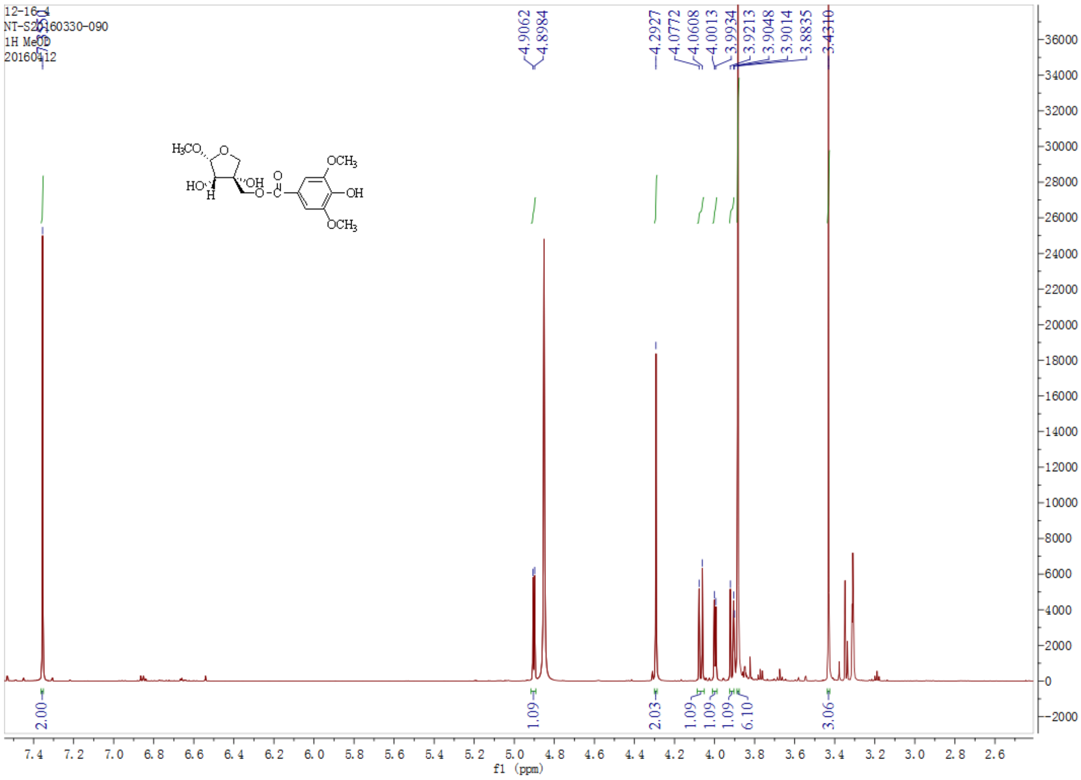

Figure S2-4 ^{1}H NMR (600 MHz, CD_3OD) spectrum of compound **2**

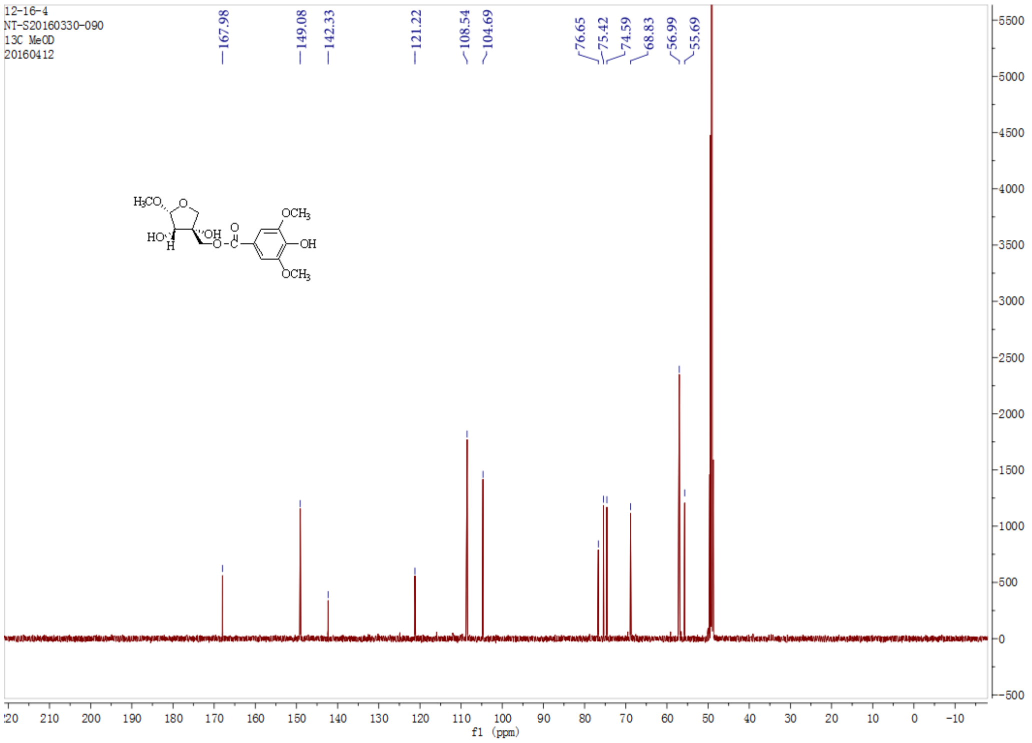

Figure S2-5 ^{13}C NMR (150 MHz, CD_3OD) spectrum of compound **2**

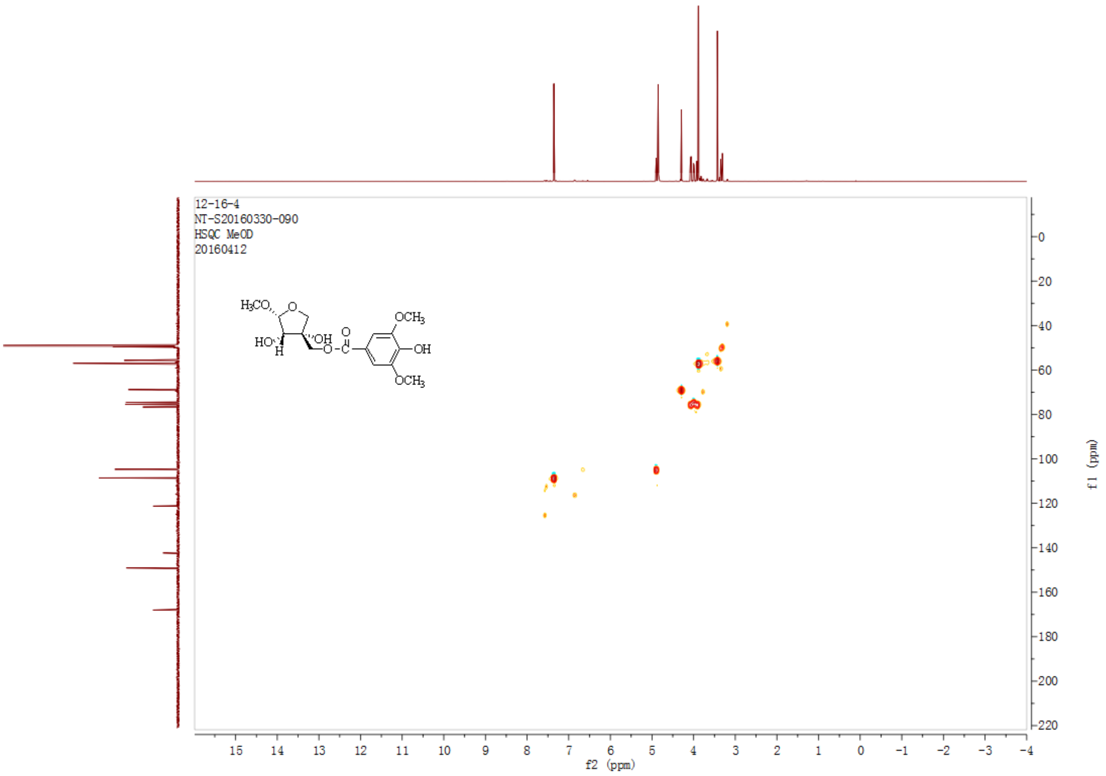

Figure S2-6 HSQC (CD_3OD) spectrum of compound **2**

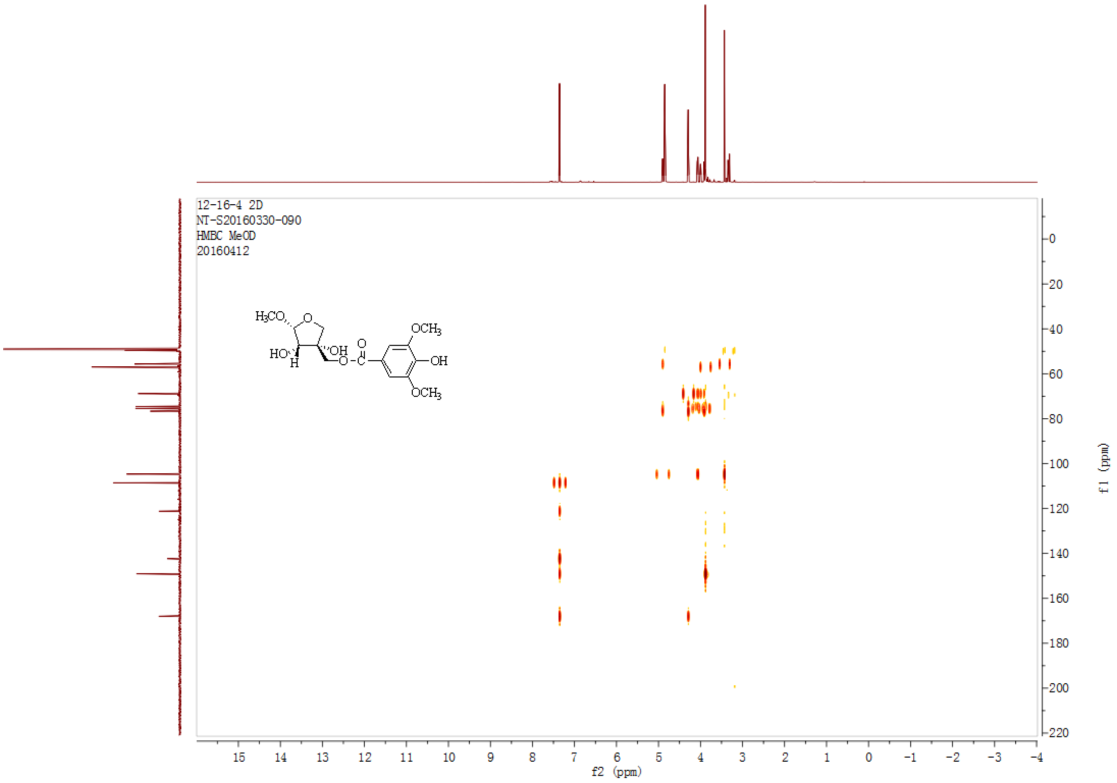

Figure S2-7 HMBC (CD_3OD) spectrum of compound **2**

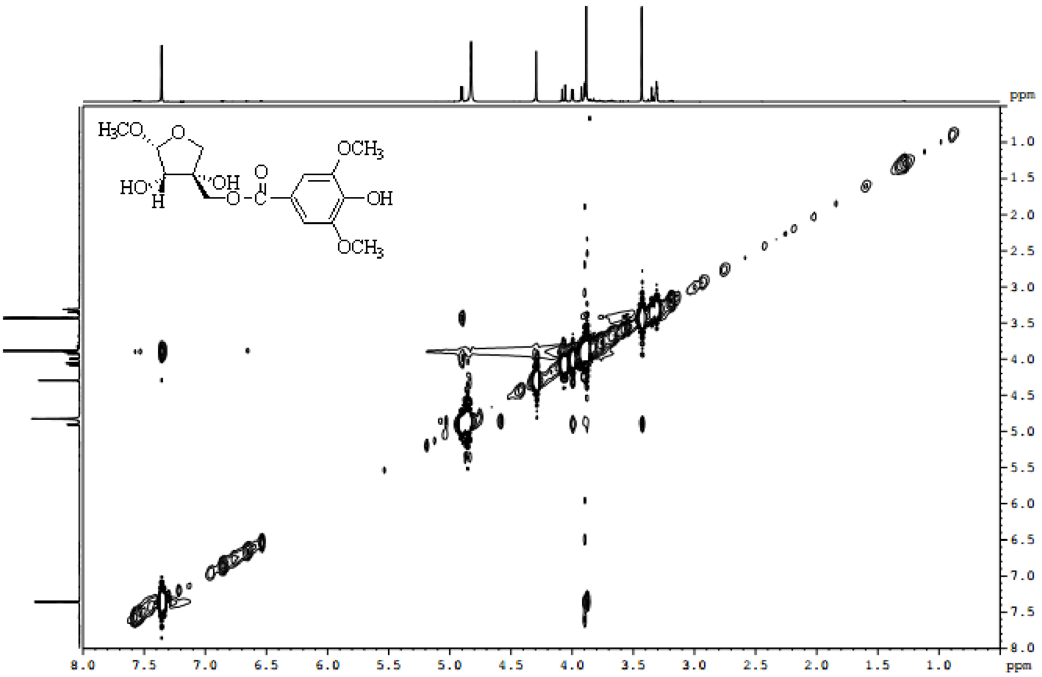

Figure S2-8 NOESY (CD_3OD) spectrum of compound **2**

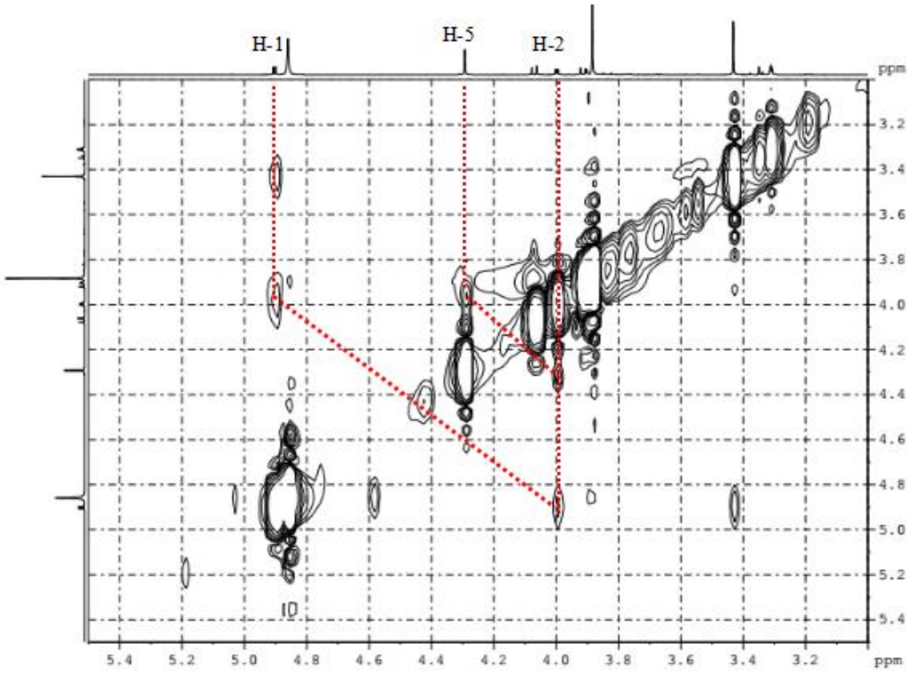

Figure S2-9 Key NOESY (CD_3OD) spectrum of compound **2**

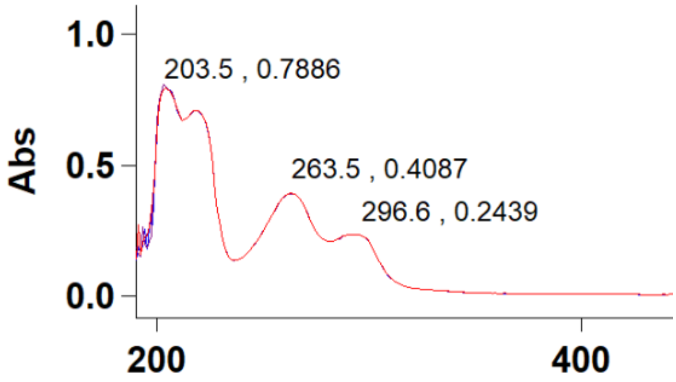

Figure S3-1 UV spectrum of compound **3** in CH3OH

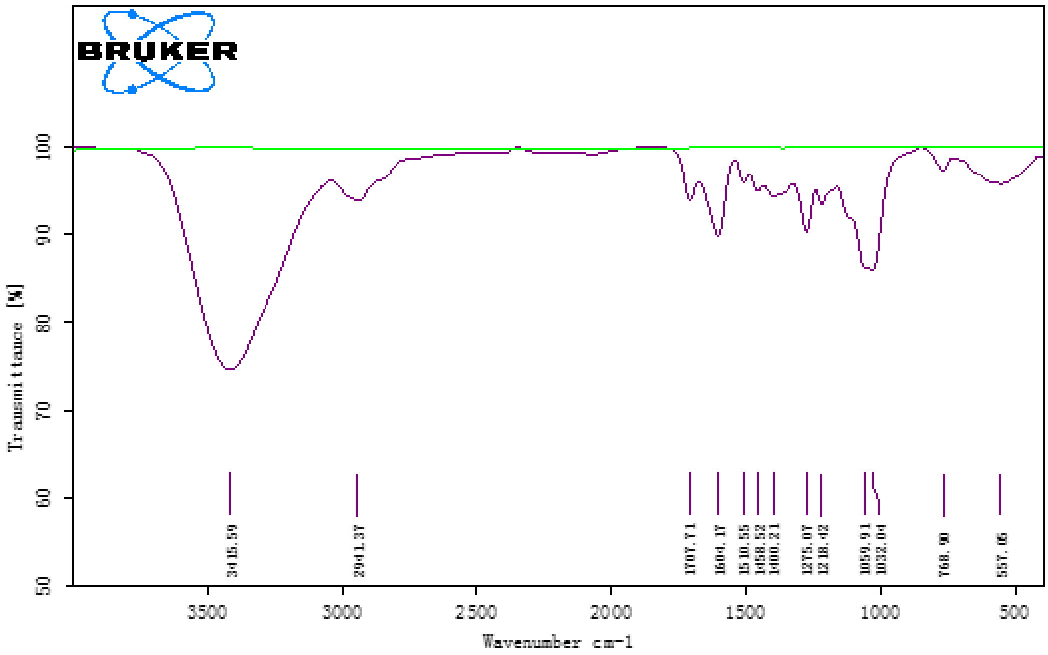

Figure S3-2 IR spectrum of compound **3** (KBr disc)

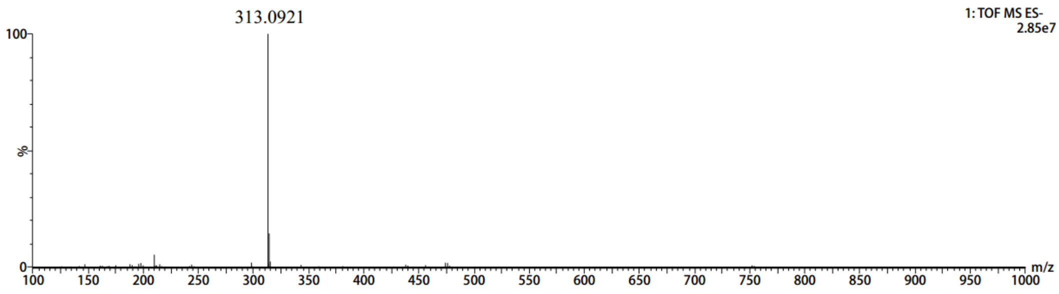

Elemental Composition Calculator

**Target m/z:**	313.0921	**Result type:**	Negative ions	**Species:**	[M-H]-
**Elements:**		C(0-20),H(0-120),O(0-10)			

Ion Formula	Calculated m/z	PPM Error
$C_{14}H_{17}O_8$	313.0923	0.64

Figure S3-3 HRESIMS of compound **3**

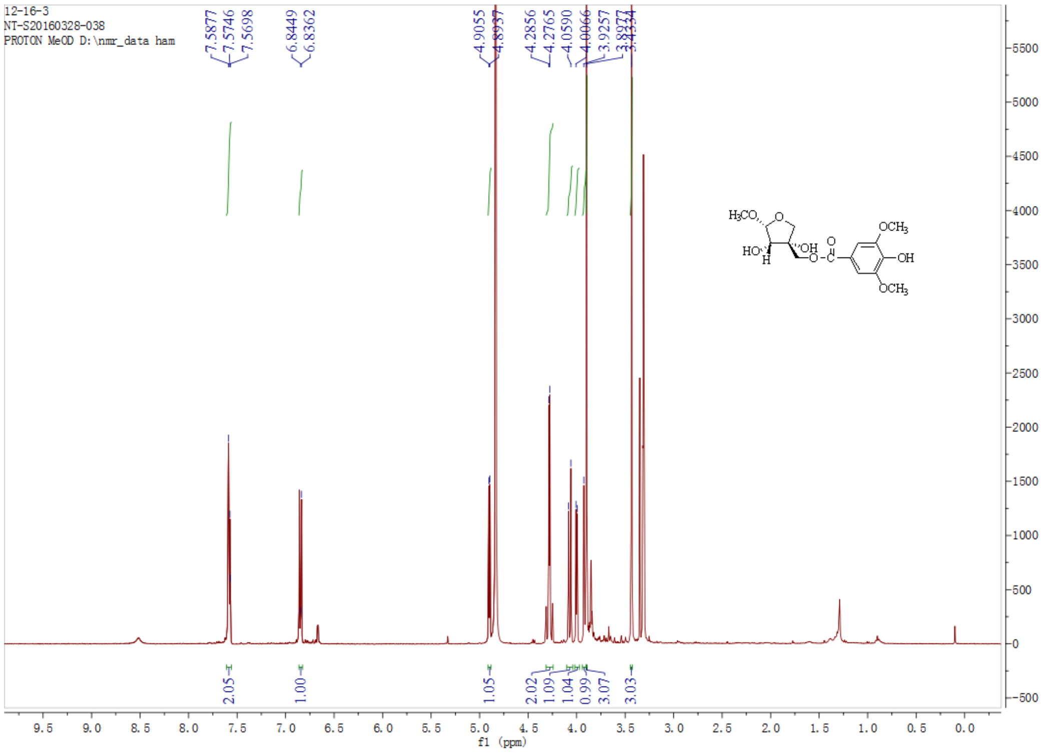

Figure S3-4 ^{1}H NMR (600 MHz, CD_3OD) spectrum of compound **3**

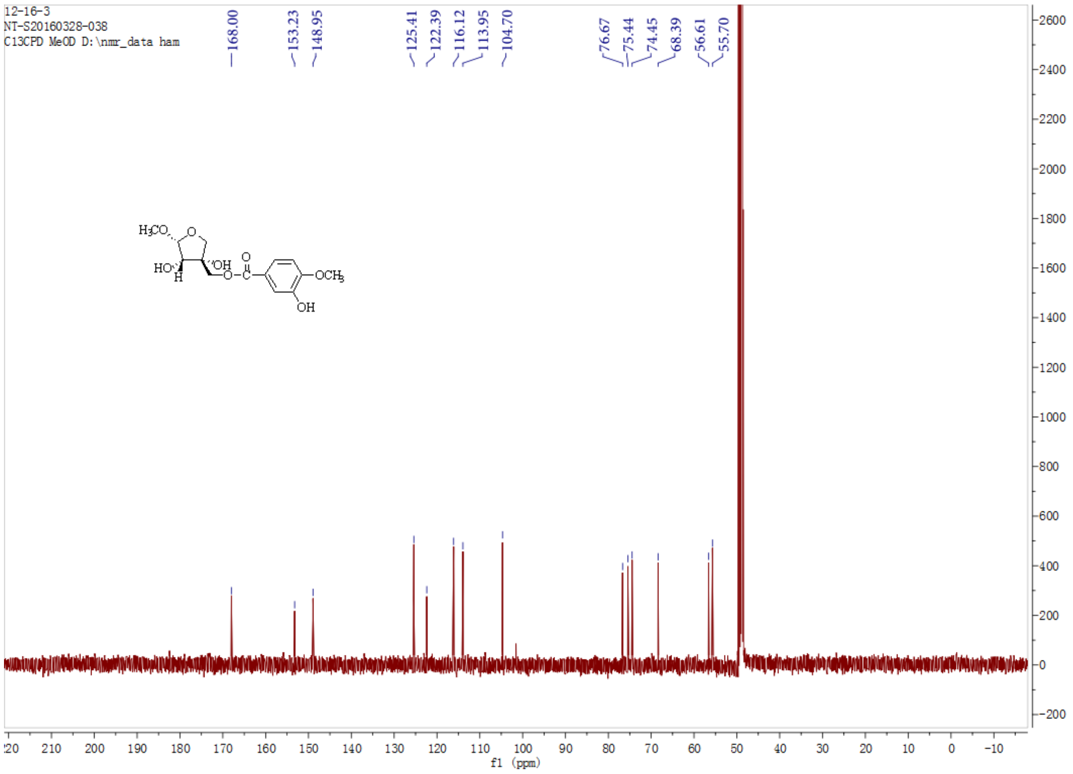

Figure S3-5 ^{13}C NMR (150 MHz, CD_3OD) spectrum of compound **3**

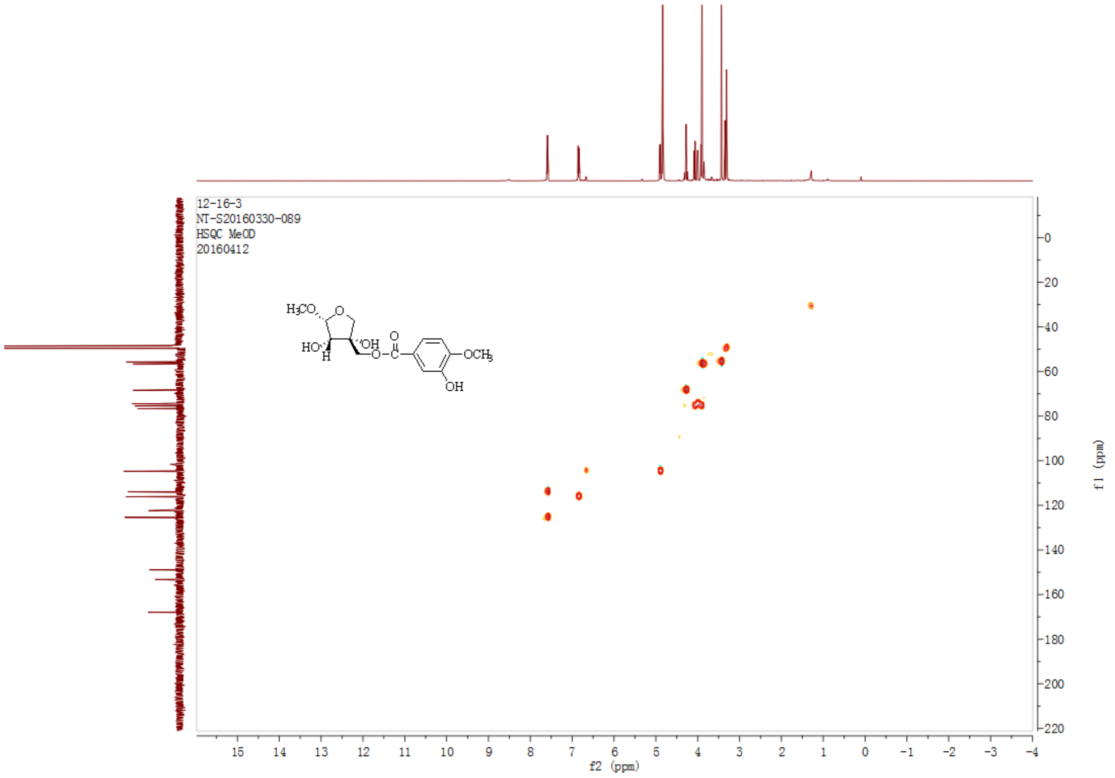

Figure S3-6 HSQC (CD_3OD) spectrum of compound **3**

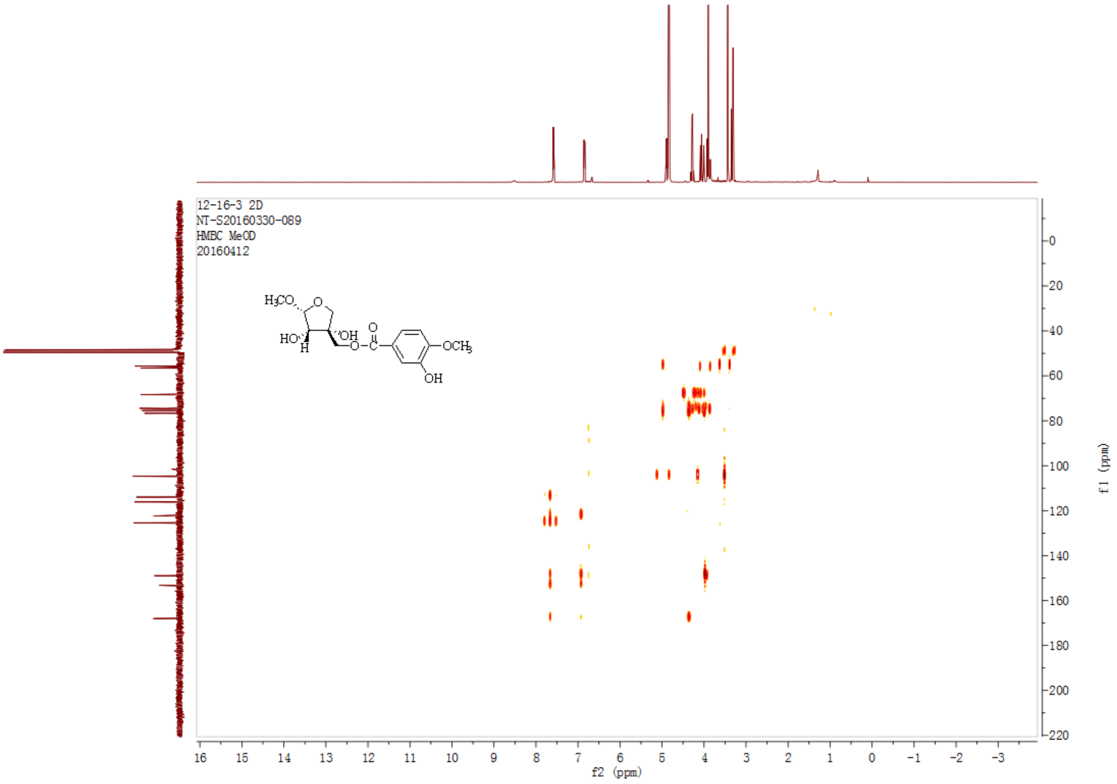

Figure S3-7 HMBC (CD_3OD) spectrum of compound **3**

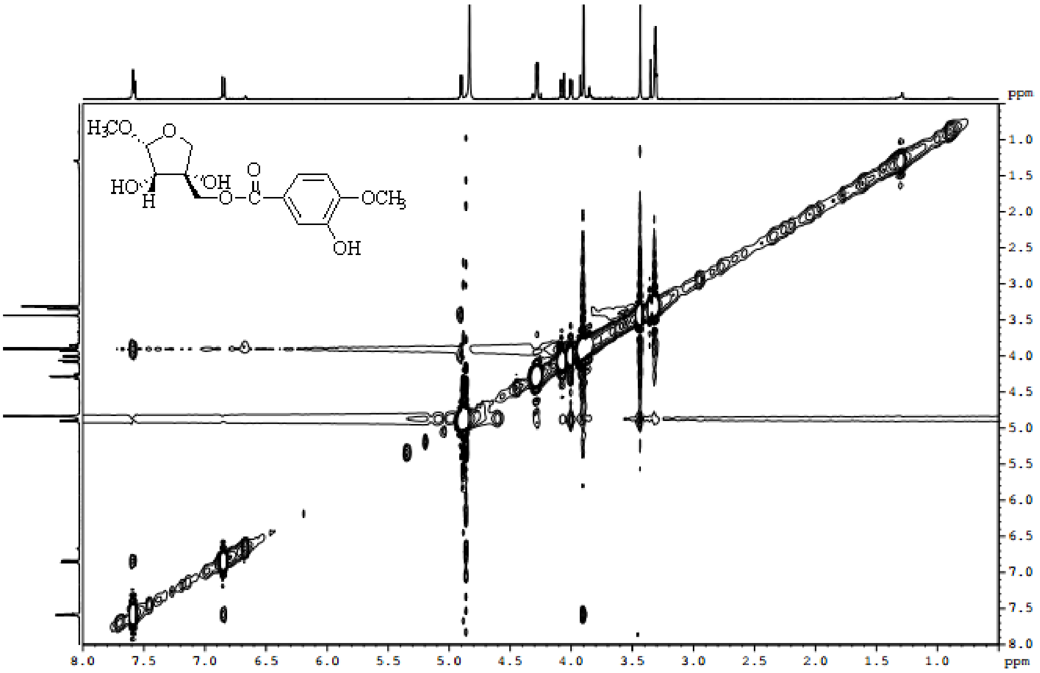

Figure S3-8 NOESY (CD_3OD) spectrum of compound **3**

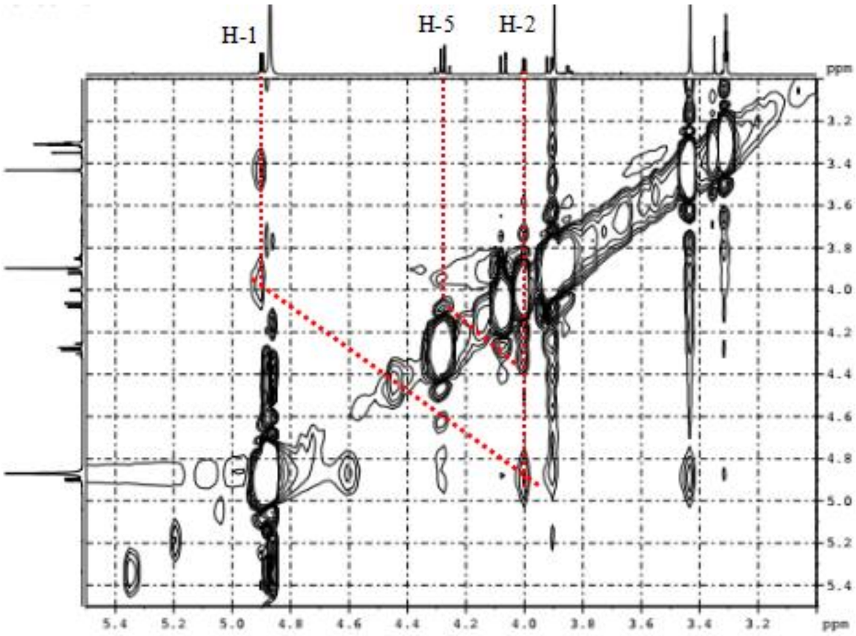

Figure S3-9 Key NOESY (CD_3OD) spectrum of compound **3**

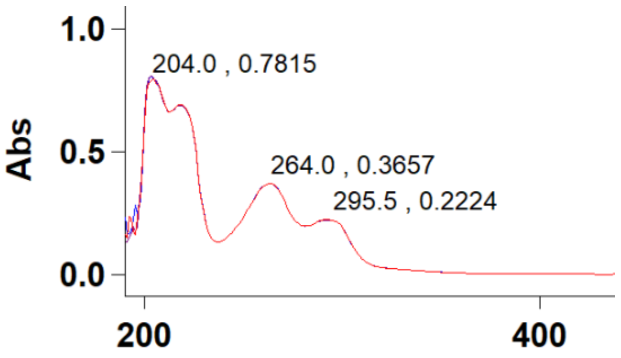

Figure S4-1 UV spectrum of compound **4** in CH_3OH

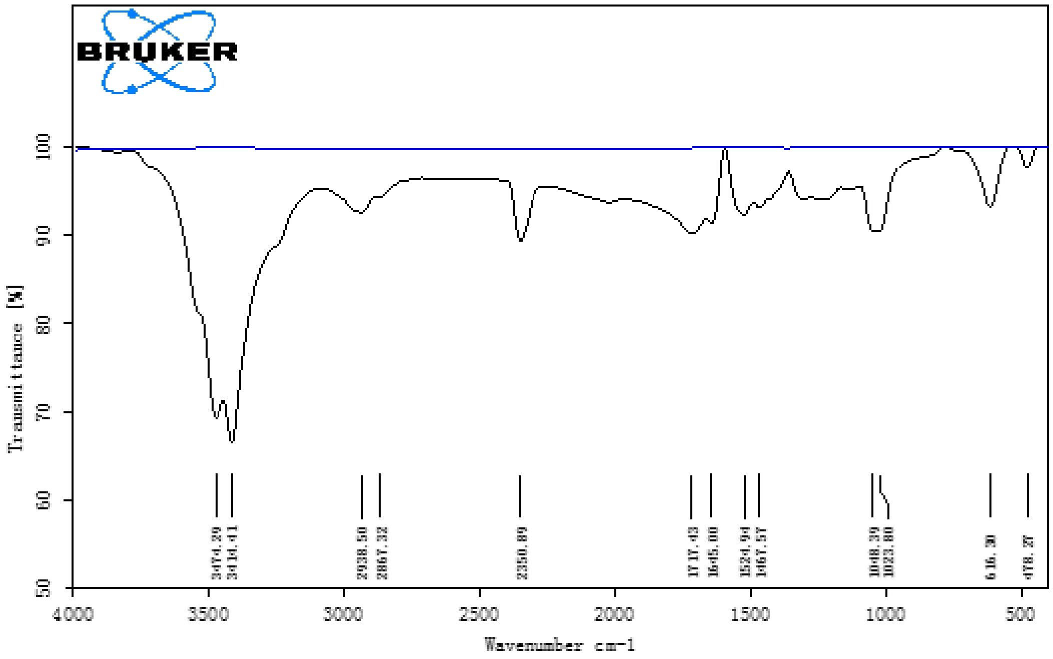

Figure S4-2 IR spectrum of compound **4** (KBr disc)

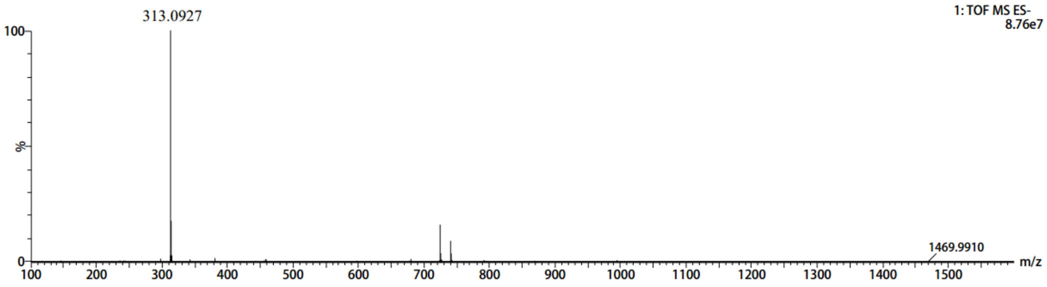

Elemental Composition Calculator

**Target m/z:**	313.0927	**Result type:**	Negative ions	**Species:**	$[M-H]^-$
**Elements:**		C(0-20),H(0-120),O(0-10)			
**Ion Formula**		**Calculated m/z**		**PPM Error**	

$C_{14}H_{17}O_8$	313.0923	1.28

Figure S4-3 HRESIMS of compound **4**

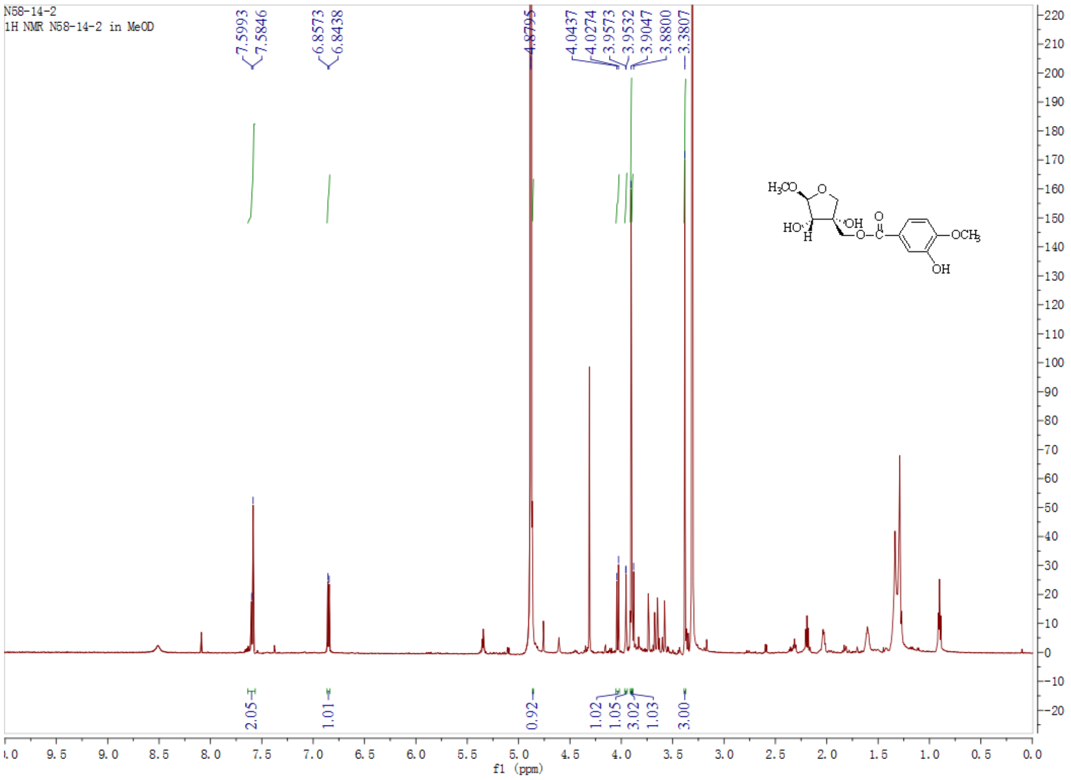

Figure S4-4 ^{1}H NMR (600 MHz, CD_3OD) spectrum of compound **4**

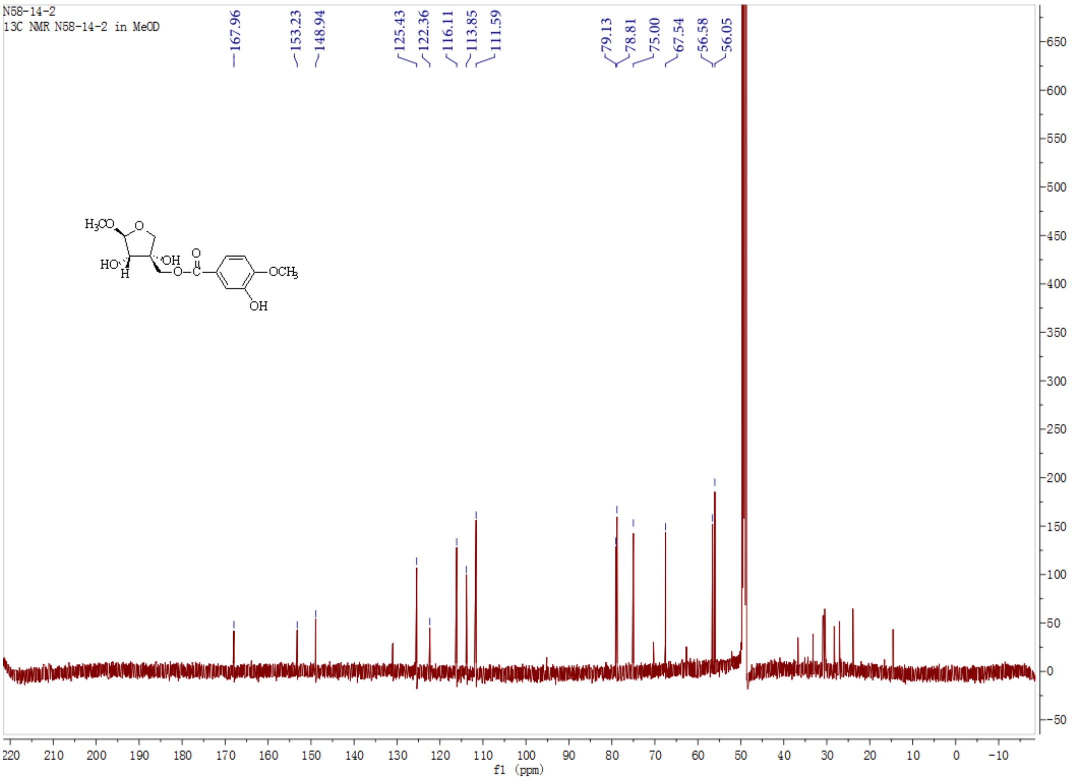

Figure S4-5 ^{13}C NMR (150 MHz, CD_3OD) spectrum of compound **4**

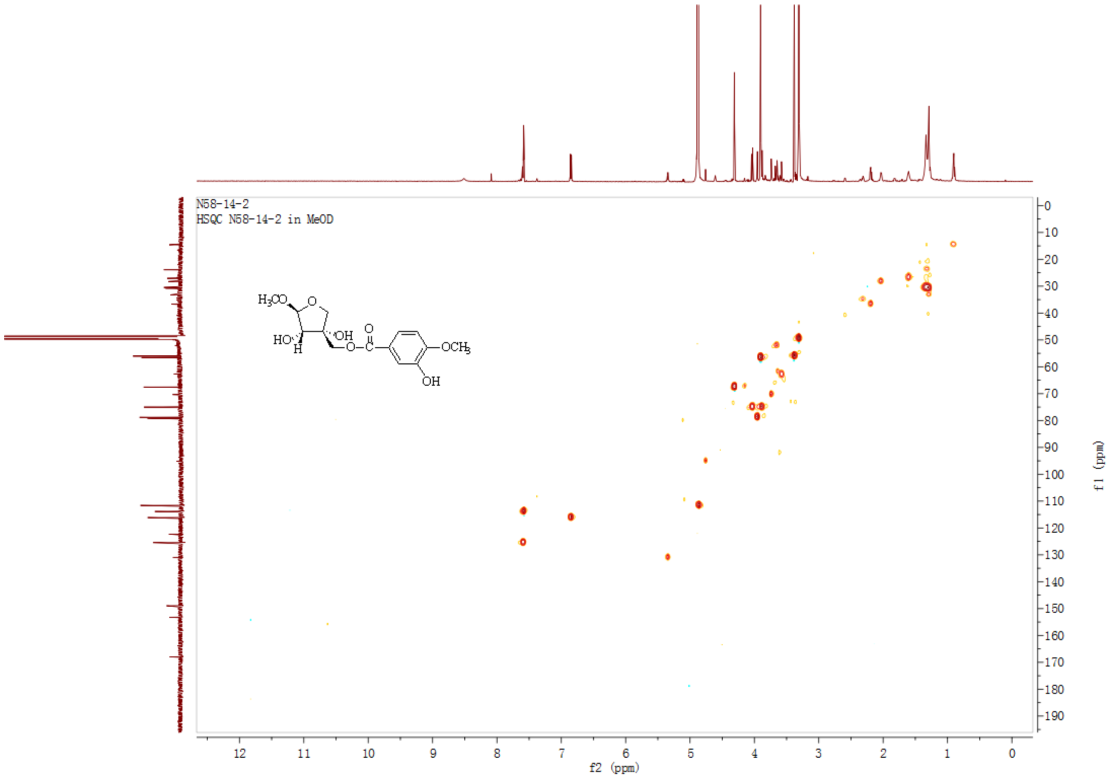

Figure S4-6 HSQC (CD_3OD) spectrum of compound **4**

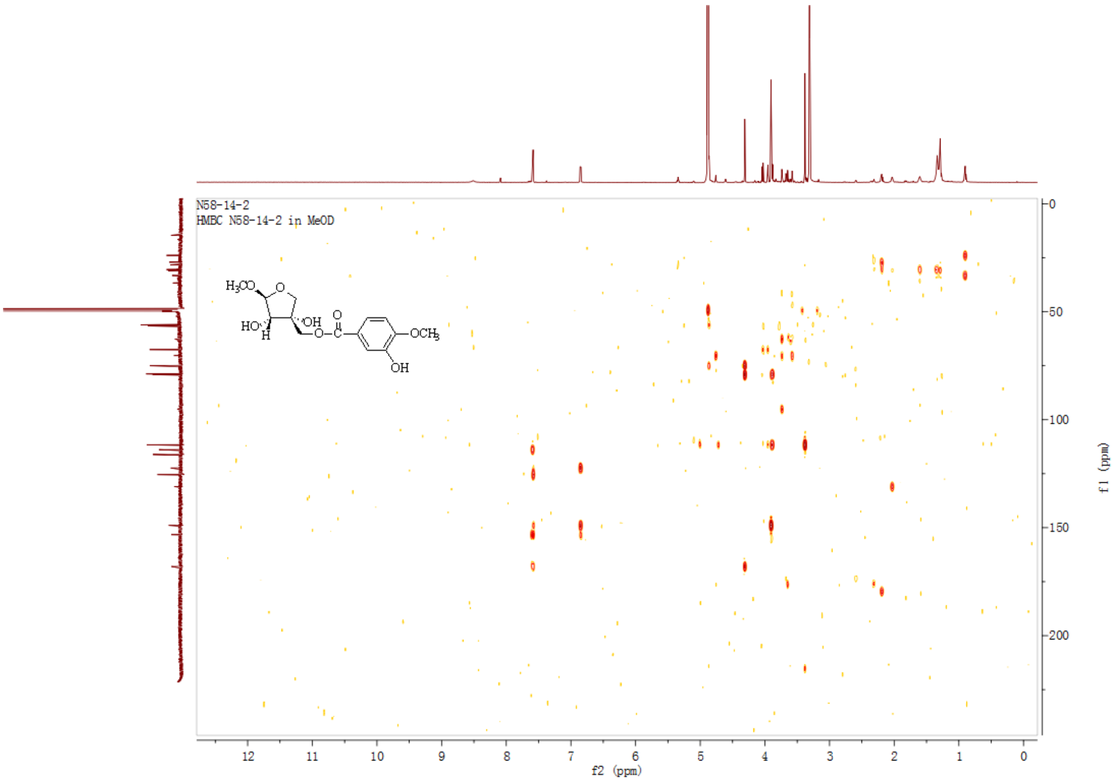

Figure S4-7 HMBC (CD_3OD) spectrum of compound **4**

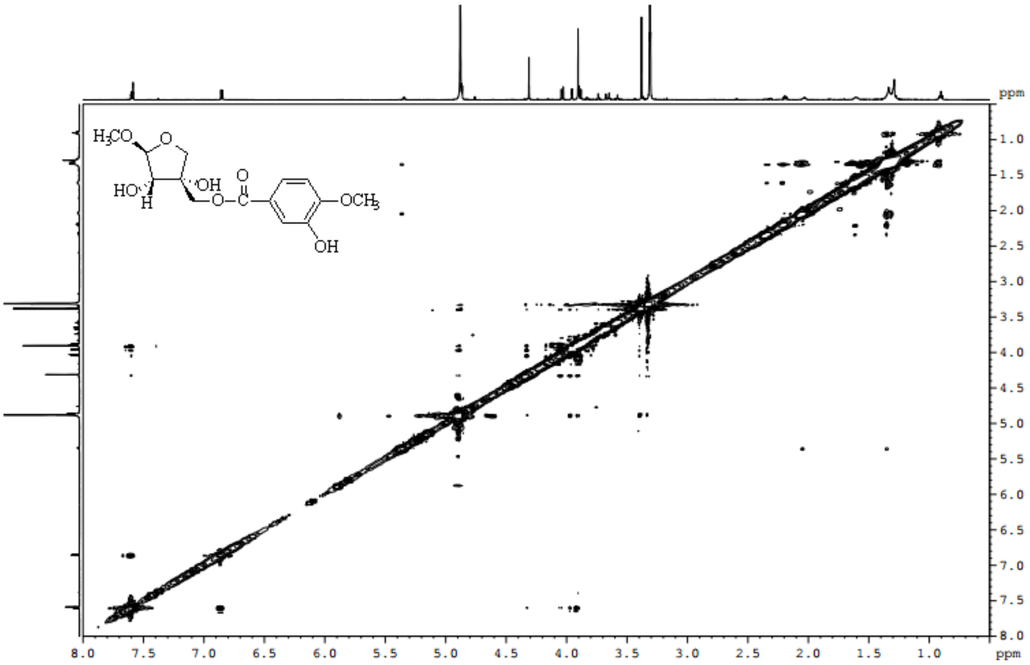

Figure S4-8 NOESY (CD_3OD) spectrum of compound **4**

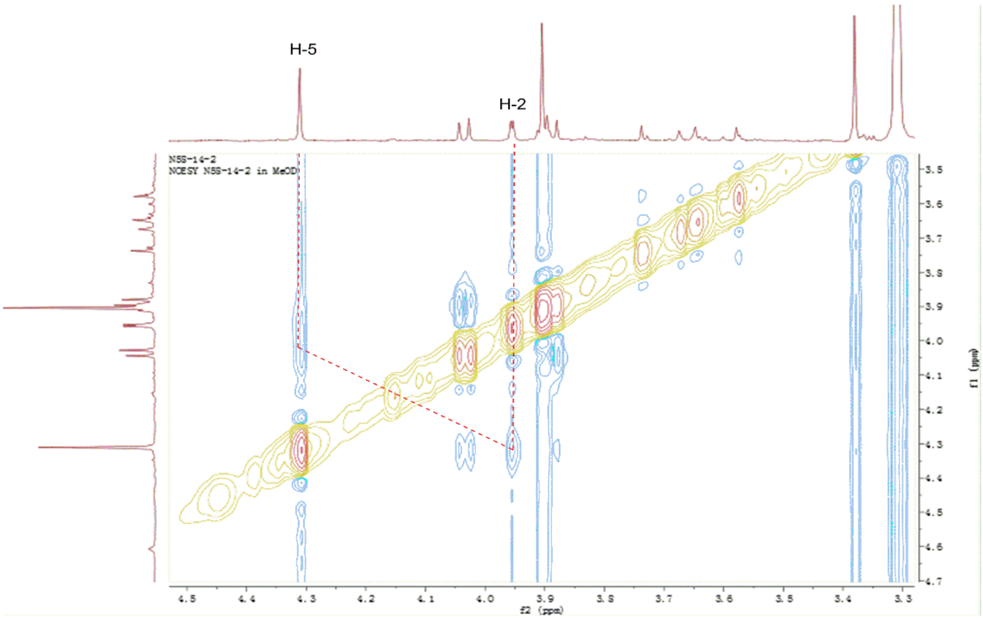

Figure S4-9 Key NOESY (CD_3OD) spectrum of compound **4**

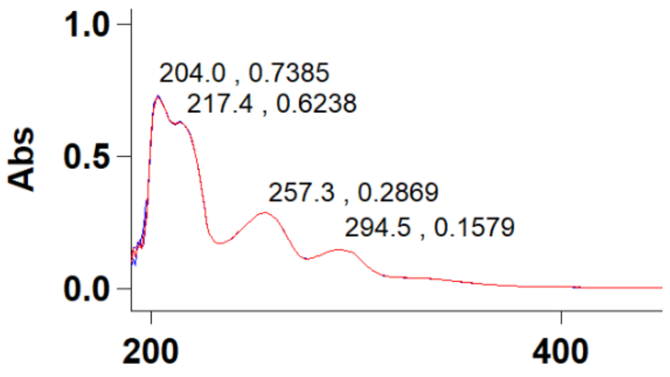

Figure S5-1 UV spectrum of compound **5** in CH_3OH

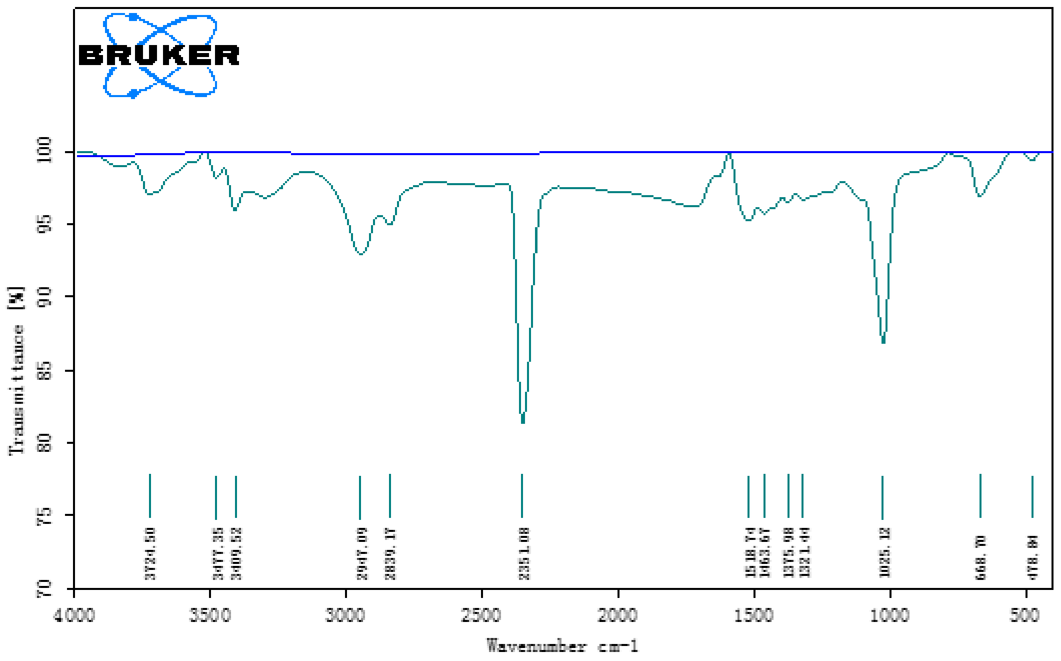

Figure S5-2 IR spectrum of compound **5** (KBr disc)

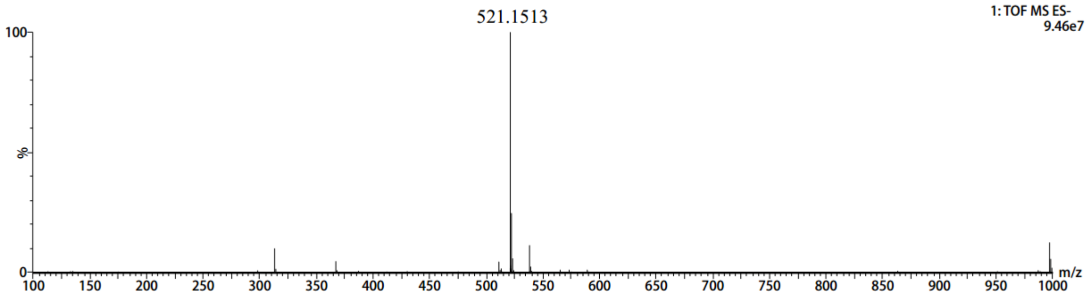

Elemental Composition Calculator

**Target m/z:**	521.1513	**Result type:**	Negative ions	**Species:**	$[M+COOH]^-$
**Elements:**		C(0-20),H(0-120),O(0-10)			

Ion Formula	Calculated m/z	PPM Error
$C_{21}H_{29}O_{15}$	521.1506	1.34

Figure S5-3 HRESIMS of compound **5**

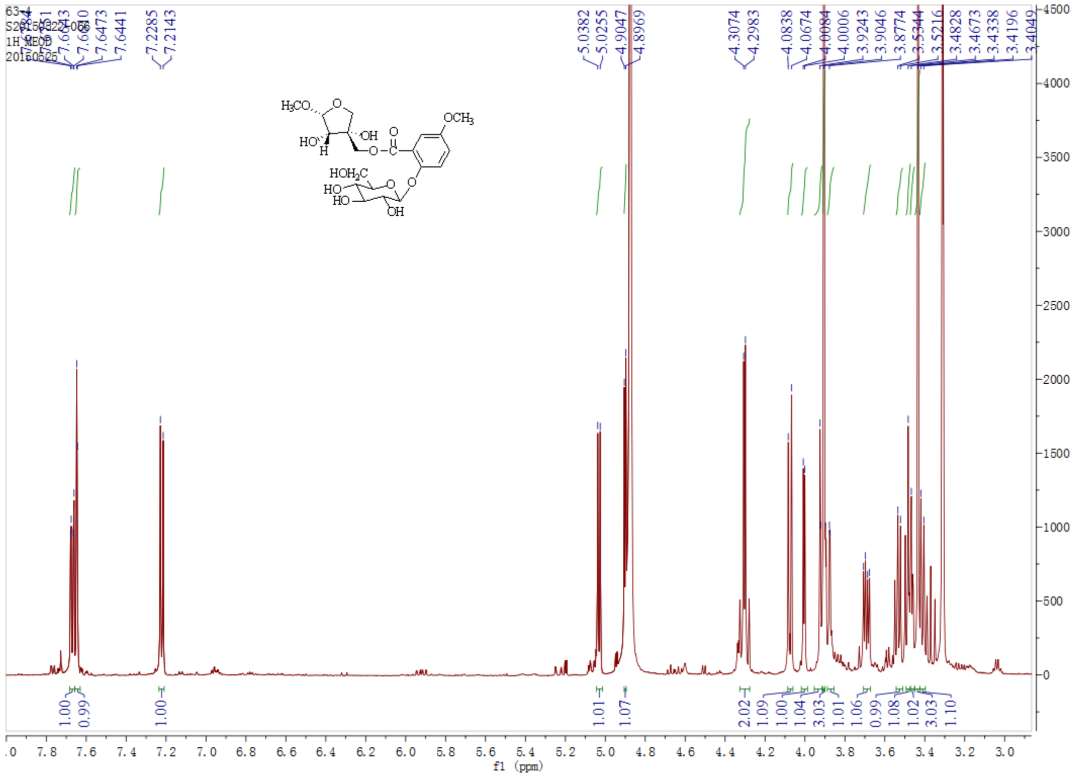

Figure S5-4 ^{1}H NMR (600 MHz, CD_3OD) spectrum of compound **5**

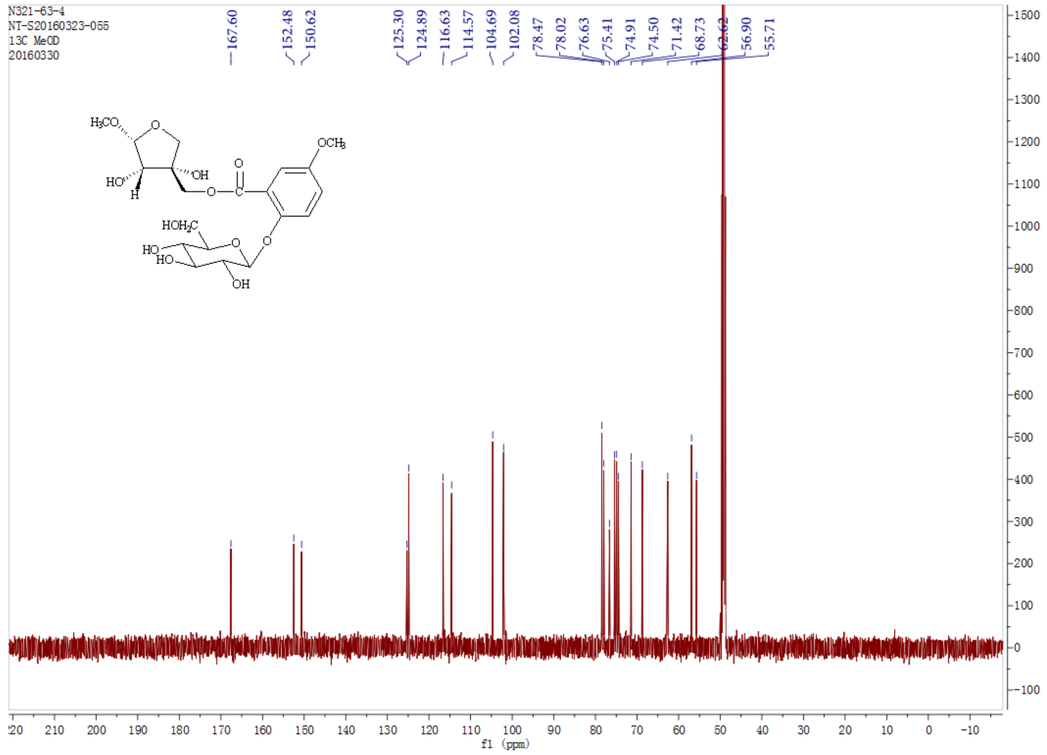

Figure S5-5 ^{13}C NMR (150 MHz, CD_3OD) spectrum of compound **5**

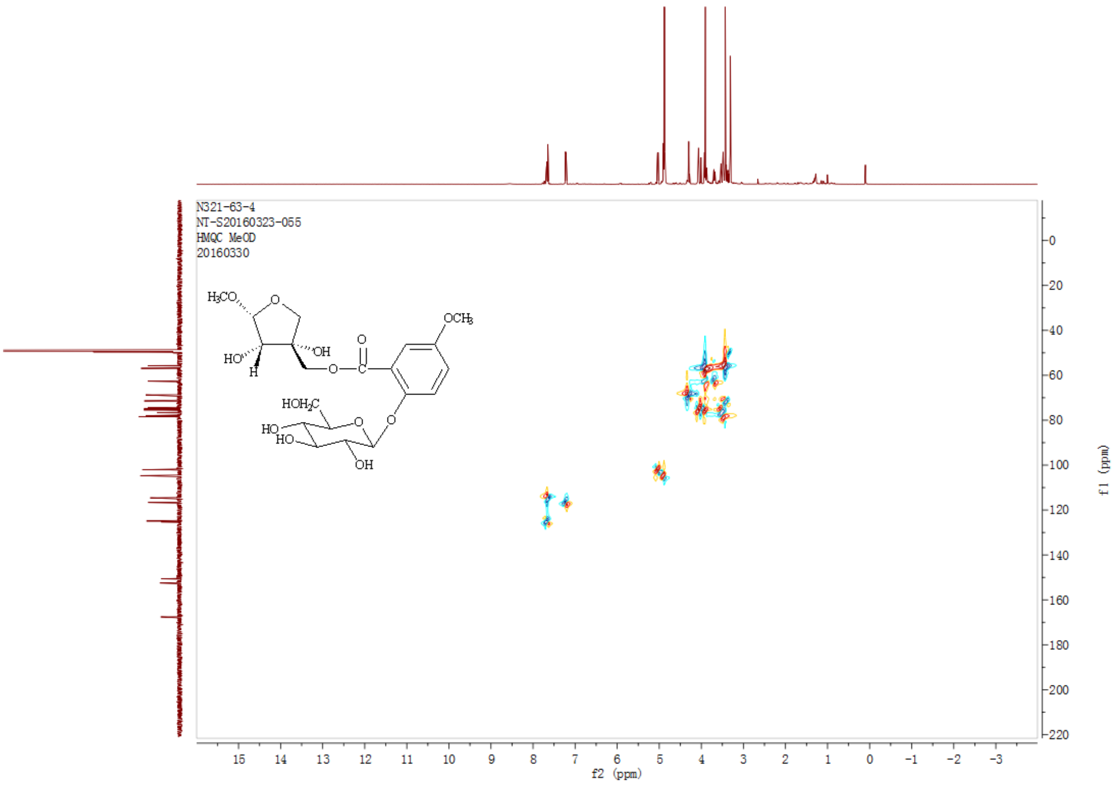

Figure S5-6 HSQC (CD_3OD) spectrum of compound **5**

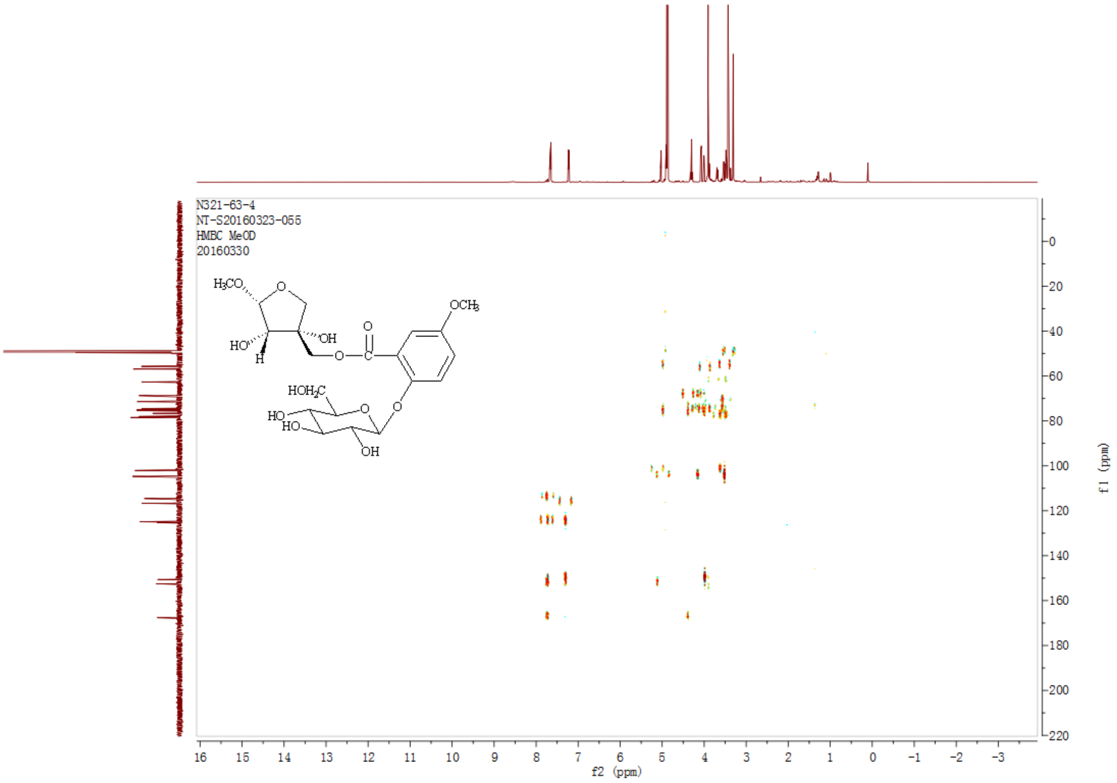

Figure S5-7 HMBC (CD_3OD) spectrum of compound **5**

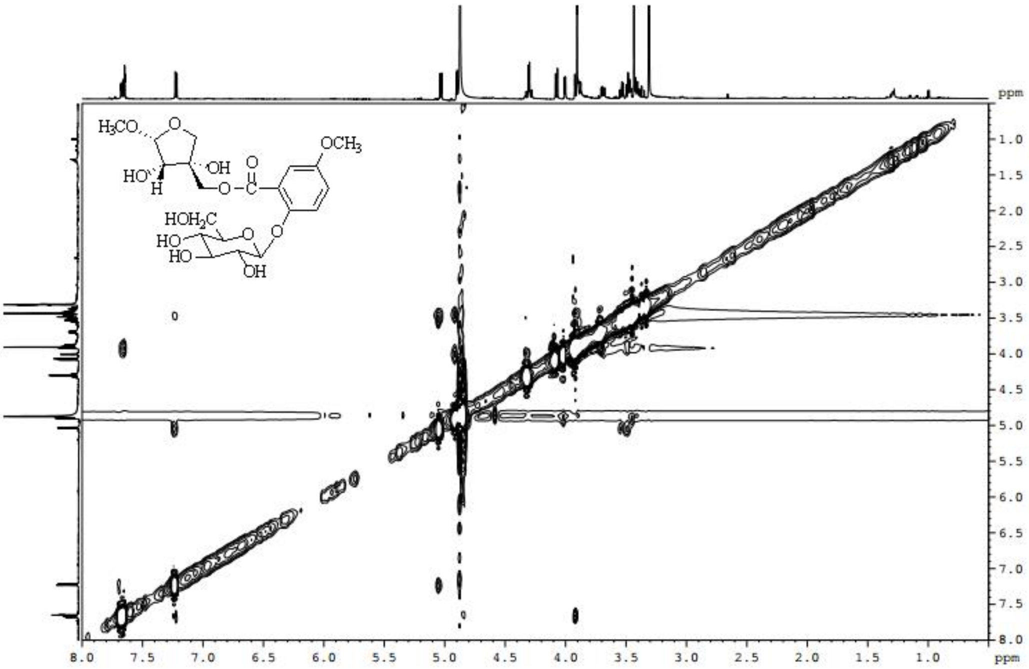

Figure S5-8 NOESY (CD_3OD) spectrum of compound **5**

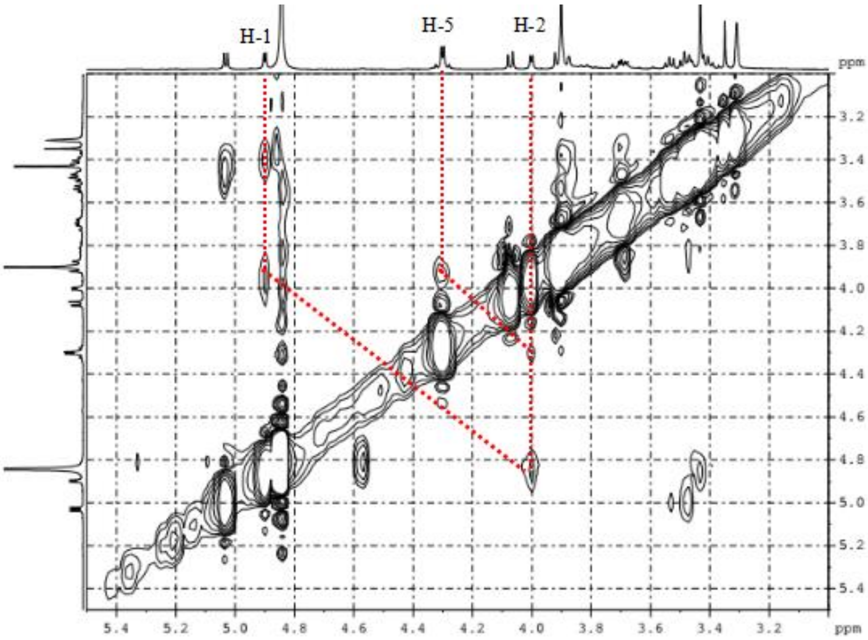

Figure S5-9 Key NOESY (CD_3OD) spectrum of compound **5**

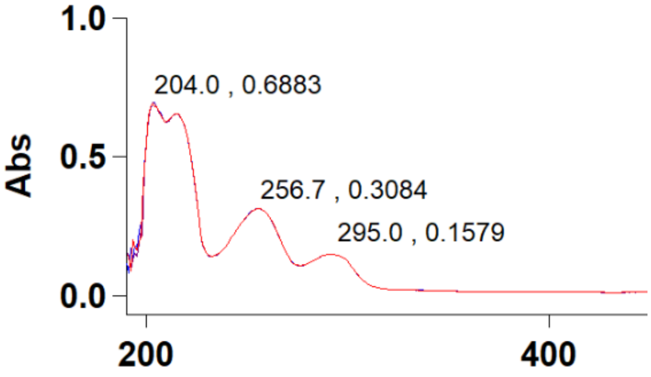

Figure S6-1 UV spectrum of compound **6** in CH_3OH

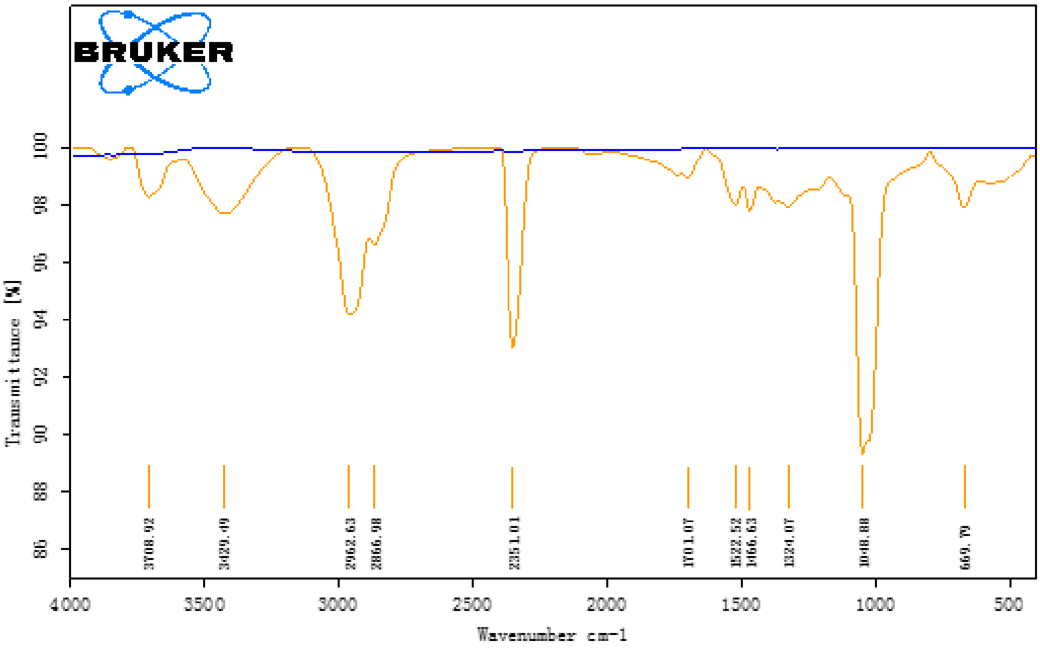

Figure S6-2 IR spectrum of compound **6** (KBr disc)

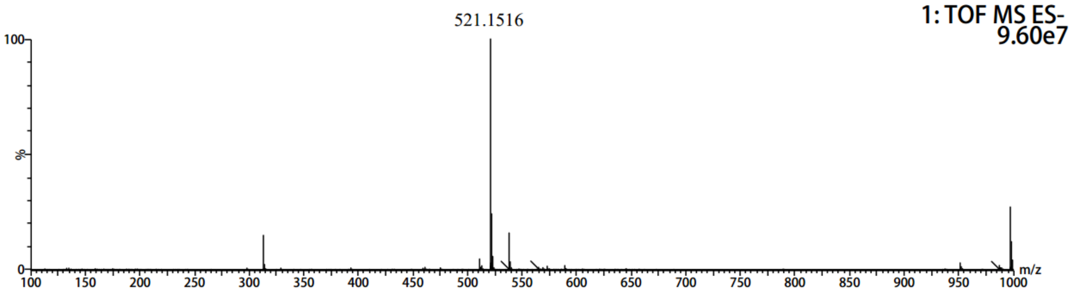

Elemental Composition Calculator

**Target m/z:**	521.1516	**Result type:**	Negative ions	**Species:**	$[M+COOH]^-$
**Elements:**		C(0-20),H(0-120),O(0-10)			

Ion Formula	Calculated m/z	PPM Error
$C_{21}H_{29}O_{15}$	521.1506	1.92

Figure S6-3 HRESIMS of compound **6**

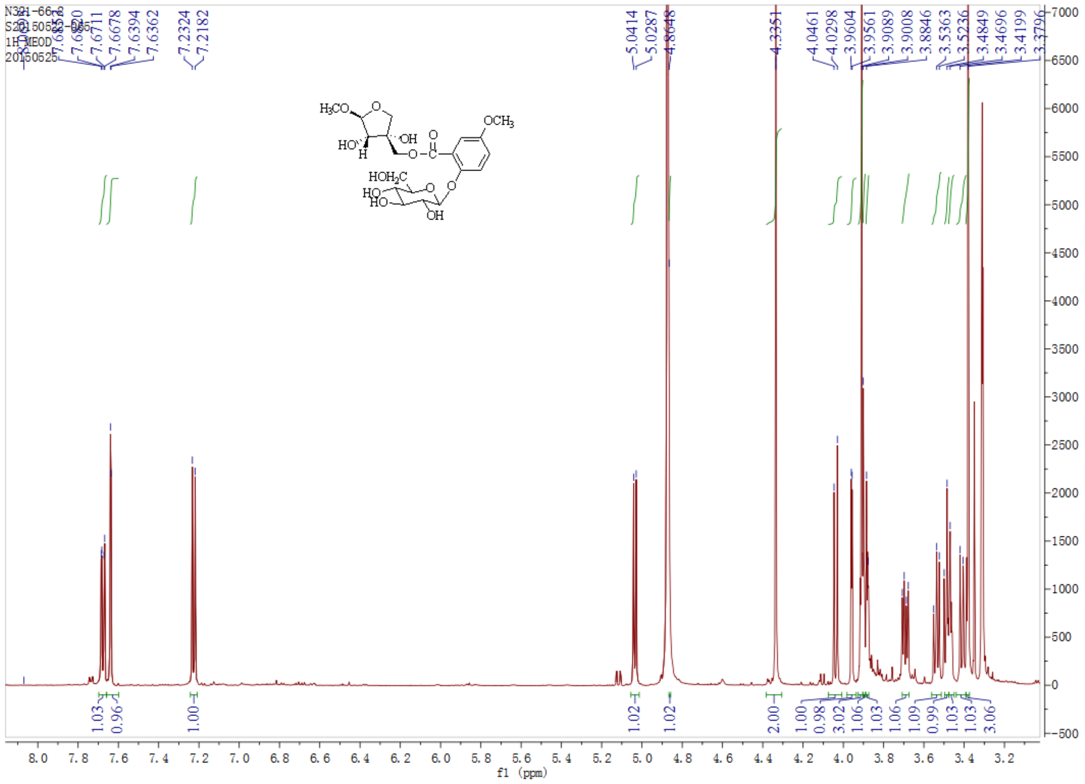

Figure S6-4 ^{1}H NMR (600 MHz, CD_3OD) spectrum of compound **6**

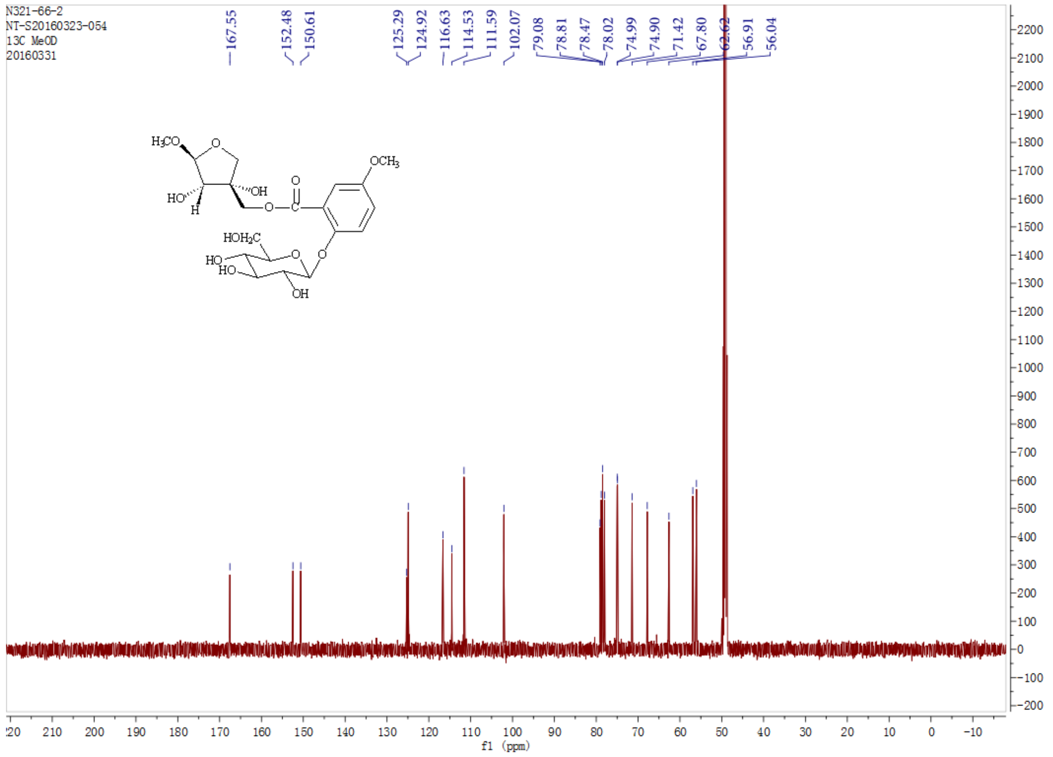

Figure S6-5 ^{13}C NMR (150 MHz, CD_3OD) spectrum of compound **6**

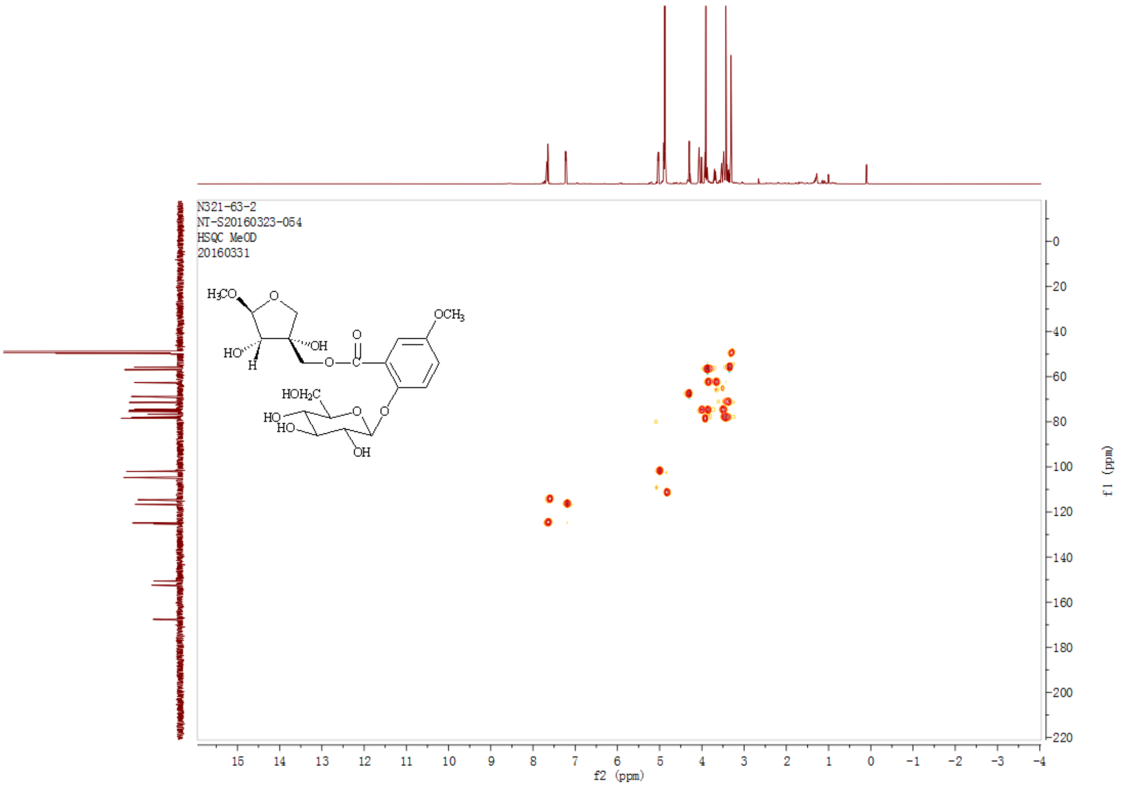

Figure S6-6 HSQC (CD_3OD) spectrum of compound **6**

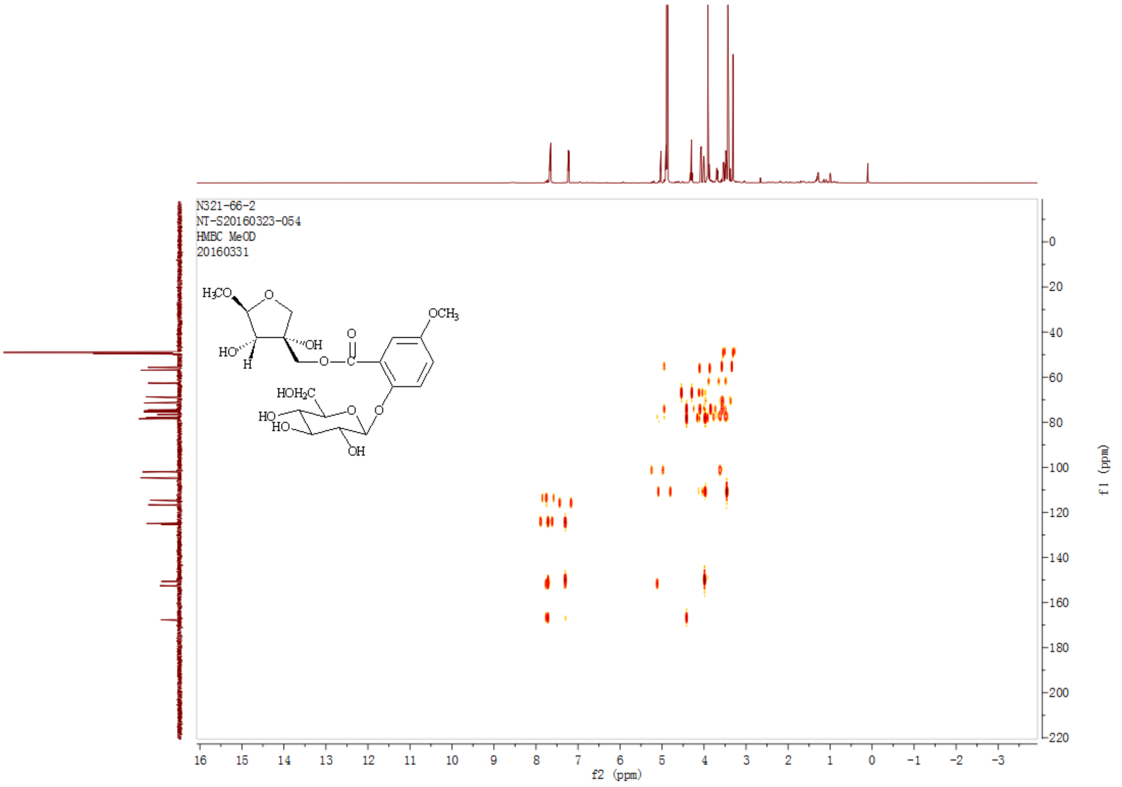

Figure S6-7 HMBC (CD_3OD) spectrum of compound **6**

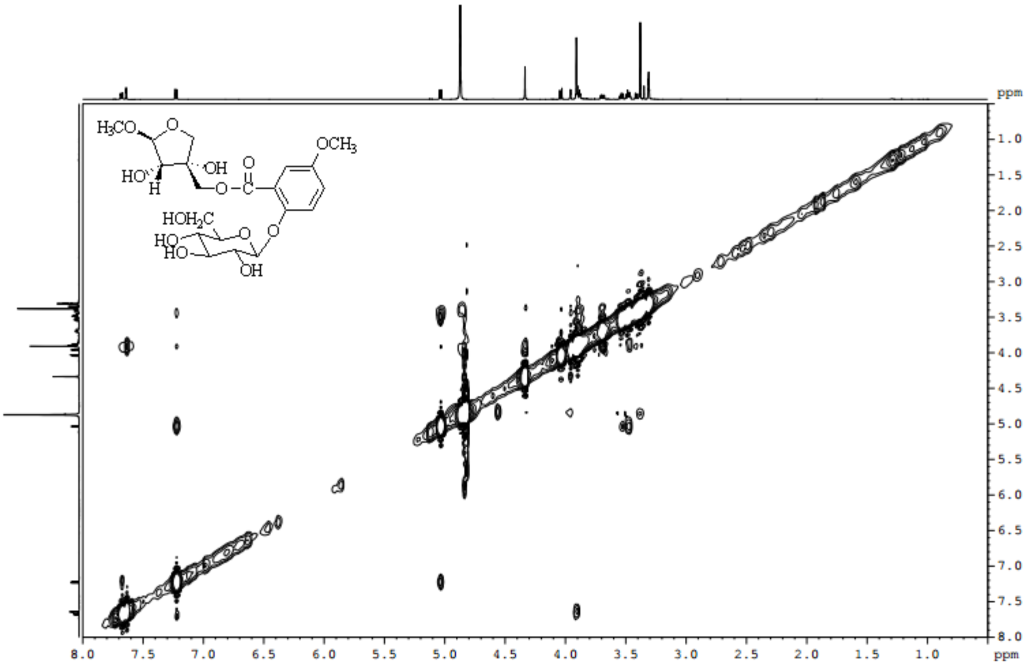

Figure S6-8 NOESY (CD_3OD) spectrum of compound **6**

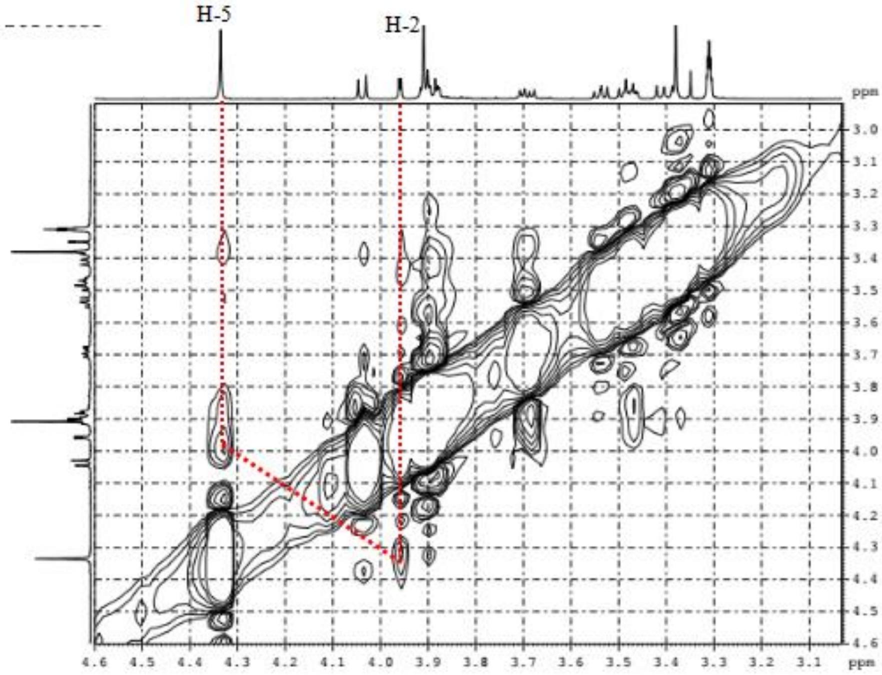

Figure S6-9 Key NOESY (CD_3OD) spectrum of compound **6**
